# Synthesis of 2-trifluoromethylpyrazolo[5,1-*a*]isoquinolines via silver triflate-catalyzed or electrophile-mediated one-pot tandem reaction

**DOI:** 10.3762/bjoc.10.238

**Published:** 2014-09-30

**Authors:** Xiaoli Zhou, Meiling Liu, Puying Luo, Yingjun Lai, Tangtao Yang, Qiuping Ding

**Affiliations:** 1 Key Laboratory of Small Functional Organic Molecule, Ministry of Education and Jiangxi's Key Laboratory of green chemistry, Jiangxi Normal University, Nanchang, Jiangxi 330022, P. R. China; 2 Department of Obstetrics and Gynecology, Jiangxi Provincial people's Hospital, Nanchang, Jiangxi 330006, P. R. China

**Keywords:** [3 + 2] cycloaddition, electrophile, *N*’-(2-alkynylbenzylidene)hydrazide, silver triflate, tandem

## Abstract

An efficient one-pot tandem cyclization/[3 + 2] cycloaddition reaction of *N*’-(2-alkynylbenzylidene)hydrazides with ethyl 4,4,4-trifluorobut-2-ynoate under silver triflate-catalyzed or electrophile-mediated conditions is described. Various trifluoromethylated pyrazolo[5,1-*a*]isoquinolines were afforded in moderate to excellent yield by this developed method.

## Introduction

Isoquinolines and isoquinoline-derived heterocycles are prevalent structural motifs in natural products and pharmaceuticals that exhibit remarkable biological activities [1–2]. Therefore, great attention has been directed toward the development of efficient methods for the selective functionalization of the isoquinoline cores. Among these, pyrazolo[5,1-*a*]isoquinoline is an important class of isoquinoline derivatives. Recently, much effort has been spent on the synthesis of these compounds due to their promising biological activities [3–18]. For instance, in 2010, Wu and co-workers found some pyrazolo[5,1-*a*]isoquinoline derivatives showing activities for the inhibition of CDC25B, TC-PTP, and PTP1B [4].

It has been proved that the physical, chemical, and biological activity of organic molecules can be dramatically improved by substitution of hydrogen with fluorine atoms because of the strong electronegativity, the small size, the strength of the C–F bond, and the low polarizability of the fluorine atom. Statistically, more than 20% of the pharmaceuticals and 40% of the agrochemicals contain one or more fluorine atoms. Thus, there has been considerable interest in developing an efficient method for the synthesis of fluorinated heterocycles. Perfluoroalkynoate is a versatile and powerful building block for generating functionalized perfluoroalkylated compounds, especially fluorinated heterocycles, by tandem reactions [19–26]. For example, 2-perfluoroalkynoates have been widely used in synthesizing fluorinated heterocycles, such as benzodiazepines [22], chromenes [21,25], and 2-oxopyridine-fused 1,3-diazaheterocycles [26].

As part of our ongoing efforts in developing synthetic approaches for the functionalization of isoquinoline cores [11,14] and the synthesis of novel fluorinated heterocycles [27] with potential biological applications, herein, we describe an efficient method for the one-pot synthesis of trifluoromethylated pyrazolo[5,1-*a*]isoquinoline derivates via a Lewis acid (AgOTf) or an electrophile- (I_2_ or ICl) promoted annulation of *N*’-(2-alkynylbenzylidene)hydrazides followed by an 1,3-dipolar cycloaddition.

## Results and Discussion

Based on Wu’s work on the silver triflate-catalyzed tandem reaction of *N*’-(2-alkynylbenzylidene)hydrazides with dimethyl acetylenedicarboxylate [28], we started our research by examining the reaction of *N*’-(2-alkynylbenzylidene)hydrazides **1a** (0.3 mmol) and ethyl 4,4,4-trifluorobut-2-ynoate (**2**, 0.6 mmol) using NaOAc (0.45 mmol) as base, in the presence of AgOTf (5 mol %) and 4 Å MS (75 mg) in CH_2_Cl_2_ (3 mL) at room temperature overnight. Surprisingly, the unexpected [3 + 2] cycloaddition product pyrazolo[5,1-*a*]isoquinoline derivative **3a** instead of the isoquinoline-based azomethine ylide [28] was obtained in good yield (84%, Table 1, entry 1). Similar yields were obtained when NaHCO_3_ or K_2_CO_3_ were used as base (83% and 86% yield, respectively, Table 1, entries 2 and 3). Several other inorganic or organic bases were examined, and the results showed that CsF was the best choice (91% yield, Table 1, entry 8). The control experiment also showed that the base was important for the reaction to proceed (8% yield, Table 1, entry 13). Subsequently, a range of solvents, such as acetonitrile, toluene, THF, dioxane and DMA were screened, and the results revealed that CH_2_Cl_2_ was the best one, and most of the others were exhibited good yields (Table 1, entry 8 and entries 14–18). The yield (65%) was reduced obviously when the loading of base was decreased to 1.0 equiv (Table 1, entry 19).

**Table 1:** Screening of conditions for the silver triflate-catalyzed reaction of *N’*-(2-alkynylbenzylidene)hydrazide **1a** with ethyl 4,4,4-trifluorobut-2-ynoate **2**^a^.

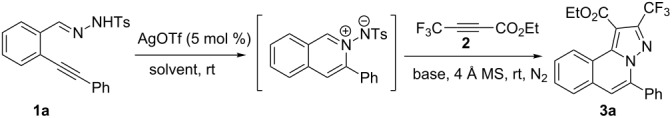			

Entry	Base (1.5 equiv)	Solvent	Yield (%)^b^

1	NaOAc	CH_2_Cl_2_	84
2	NaHCO_3_	CH_2_Cl_2_	83
3	K_2_CO_3_	CH_2_Cl_2_	86
4	Cs_2_CO_3_	CH_2_Cl_2_	63
5	NaOH	CH_2_Cl_2_	51
6	*t*-BuOK	CH_2_Cl_2_	trace
7	KF	CH_2_Cl_2_	80
8	CsF	CH_2_Cl_2_	91
9	DABCO	CH_2_Cl_2_	60
10	NEt_3_	CH_2_Cl_2_	61
11	DBU	CH_2_Cl_2_	50
12	Pyridine	CH_2_Cl_2_	37
13	–	CH_2_Cl_2_	8
14	CsF	CH_3_CN	65
15	CsF	toluene	69
16	CsF	THF	71
17	CsF	dioxane	76
18	CsF	DMA	87
19	CsF (1.0 equiv)	CH_2_Cl_2_	65

^a^Reaction conditions: *N*’-(2-alkynylbenzylidene)hydrazide **1a** (0.3 mmol), AgOTf (5 mol %), solvent (3 mL), ethyl 4,4,4-trifluorobut-2-ynoate (**2**, 0.6 mmol, 2.0 equiv), base (1.5 equiv), 4 Å MS (75 mg), rt, overnight. ^b^Isolated yield based on **1a**.

**Figure 1:**
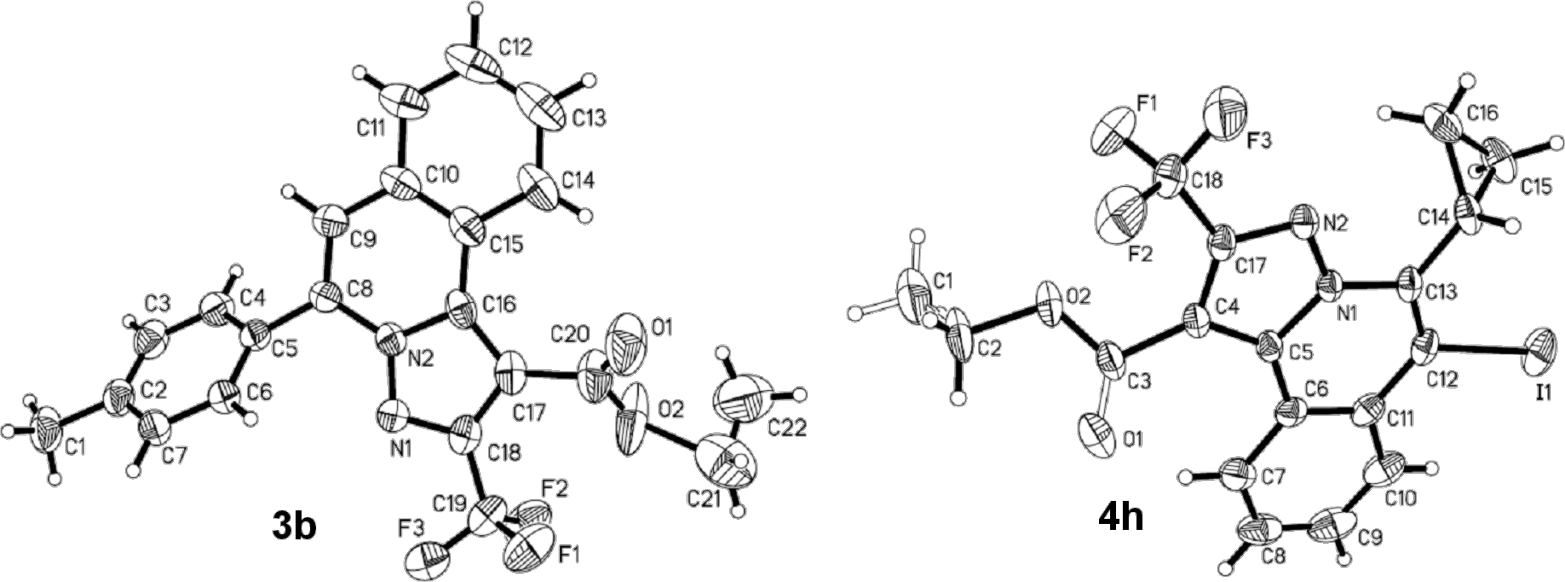
ORTEP diagrams of **3b** and **4h**.

To explore the scope of this tandem cyclization/[3 + 2] cycloaddition reaction, a range of *N*’-(2-alkynylbenzylidene)hydrazides **1a–j** were prepared from the corresponding aldehydes and applied to the synthesis of trifluoromethylated pyrazolo[5,1-*a*]isoquinoline derivatives **3** under the optimized conditions (Table 1, entry 8). As shown in Table 2, for most cases, *N*’-(2-alkynylbenzylidene)hydrazides **1** reacted with ethyl 4,4,4-trifluorobut-2-ynoate **2** affording the corresponding products **3** in good to excellent yields. For instance, substrate **1b** bearing an electron-donating substituent (methyl) reacted with **2** under the present reaction conditions gave the desired product **3b** in good yield (86%, Table 2, entry 2). The structure of **3b** was verified by ^1^H and ^13^C NMR, HRMS, as well as X-ray diffraction analysis (Figure 1, for details, see Supporting Information File 1). As expected, the substrates **1e–h** with electron-withdrawing substituents are suitable partners in this process and the corresponding pyrazolo[5,1-*a*]isoquinolines **3e–h** were obtained in good yields. Fortunately, alkyl-substituted *N*’-(2-alkynylbenzylidene)hydrazide was demonstrated to be good partner in the transformation. For instance, N’-(2-alkynylbenzylidene)hydrazide **1i** reacted with **2**, leading to the desired pyrazolo[5,1-a]isoquinoline **3i** in 90% yield (Table 2, entry 9).

**Table 2:** Silver triflate-catalyzed tandem reactions of *N*’-(2-alkynylbenzylidene)hydrazides **1** with ethyl 4,4,4-trifluorobut-2-ynoate **2**.

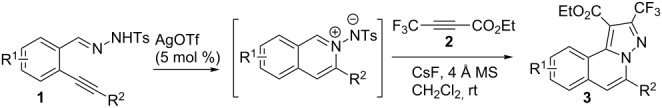							

Entry	R^1^, R^2^**/1**	Product **3**	Yield (%)^a^	Entry	R^1^, R^2^**/1**	Product **3**	Yield (%)^a^

1	R^1^ = H R^2^ = Ph **1a**	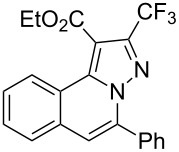 **3a**	91	6	R^1^ = H R^2^ = 4-FC_6_H_4_ **1f**	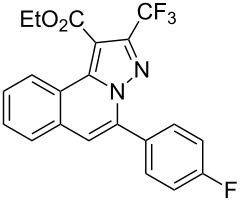 **3f**	80
2	R^1^ = H R^2^ = 4-MeC_6_H_4_ **1b**	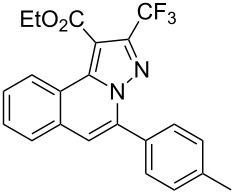 **3b**	86	7	R^1^ = H R^2^ = 4-AcC_6_H_4_ **1g**	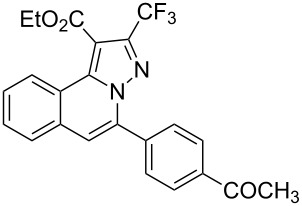 **3g**	87
3	R^1^ = H R^2^ = 4-MeOC_6_H_4_ **1c**	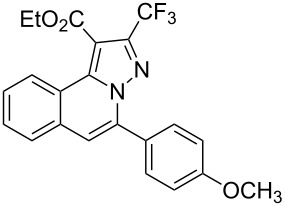 **3c**	75	8	R^1^ = H R^2^ = 4-NO_2_C_6_H_4_ **1h**	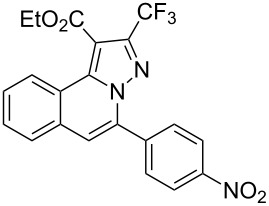 **3h**	47
4	R^1^ = H R^2^ = 4-EtOC_6_H_4_ **1d**	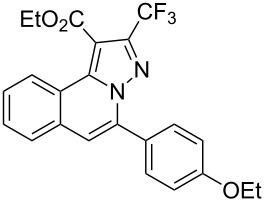 **3d**	89	9	R^1^ = H R^2^ = cyclopropyl **1i**	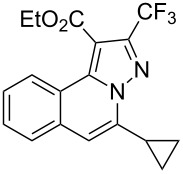 **3i**	90
5	R^1^ = H R^2^ = 4-ClC_6_H_4_ **1e**	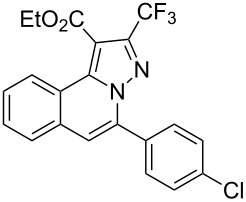 **3e**	85	10	R^1^ = 3-F R^2^ = Ph **1j**	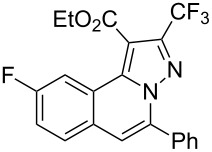 **3j**	44

^a^Isolated yields based on *N*’-(2-alkynylbenzylidene)hydrazides **1**.

Subsequently, based on our previous reports on electrophile-mediated electrophilic cyclization reaction [28–29], one-pot tandem electrophilic cyclization/[3 + 2] cycloaddition of *N*’-(2-alkynylbenzylidene)hydrazides **1**, electrophiles (I_2_ or ICl), and ethyl 4,4,4-trifluorobut-2-ynoate (**2**) were carried out under mild conditions. The results are summarized in Table 3. For all cases, this tandem reaction worked well leading to the corresponding iodinated fluorine-containing pyrazolo[5,1-*a*]isoquinolines **4** in moderate to excellent yields. Various functional groups, such as methyl, methoxy, ethoxy, halogen, acetyl, nitro, and cyclopropyl groups were tolerated under the reaction conditions. In general, substrates bearing electron-donating substituents show better reactivity than those with electron-withdrawing substituents. For instance, methyl-substituted *N*’-(2-alkynylbenzylidene)hydrazide **1b** reacted with iodine and ethyl 4,4,4-trifluorobut-2-ynoate (**2**) in the presence of CsF and 4 Å MS leading to the desired product **4b** in 90% yield (Table 3, entry 3). A relatively lower yield was obtained when nitro substituted *N*’-(2-alkynylbenzylidene)hydrazide **1h** was used, and the desired product **4g** was obtained in 50% yield (Table 3, entry 9). Alkyl-substituted product **4h** was obtained in good yields when substrate **1i** reacted with iodine or ICl (Table 3, entries 10 and 11), the structure of **4h** was verified by ^1^H and ^13^C NMR, HRMS, as well as X-ray diffraction analysis (Figure 1, for details, see Supporting Information File 1). Based on this one-pot tandem electrophilic cyclization/[3 + 2] cycloaddition reactions, highly functionalized pyrazolo[5,1-*a*]isoquinolines can be obtained via palladium-catalyzed cross-coupling reaction.

**Table 3:** One-pot tandem reactions of *N*’-(2-alkynylbenzylidene)hydrazides **1**, electrophiles, and ethyl 4,4,4-trifluorobut-2-ynoate (**2**)^a^.

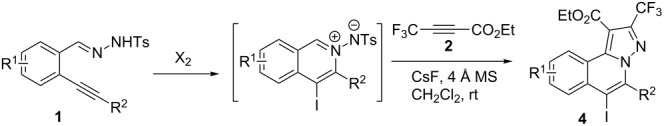									

Entry	**1**	X_2_	**4**	Yield (%)^b^	Entry	**1**	X_2_	**4**	Yield (%)^b^

1	**1a**	I_2_	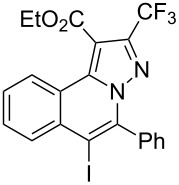 **4a**	71	7	**1f**	I_2_	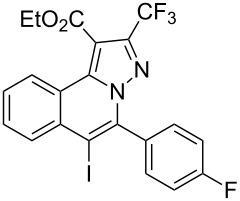 **4e**	62
2	**1a**	ICl	**4a**	78	8	**1g**	I_2_	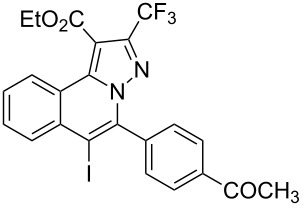 **4f**	84
3	**1b**	I_2_	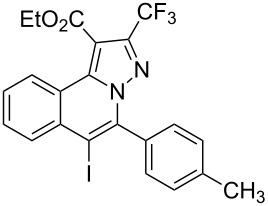 **4b**	90	9	**1h**	ICl	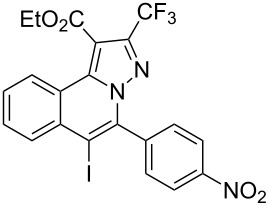 **4g**	50
4	**1b**	ICl	**4b**	82	10	**1i**	I_2_	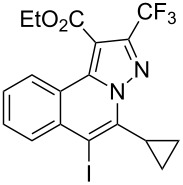 **4h**	85
5	**1c**	I_2_	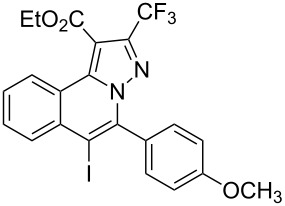 **4c**	80	11	**1i**	ICl	**4h**	62
6	**1d**	I_2_	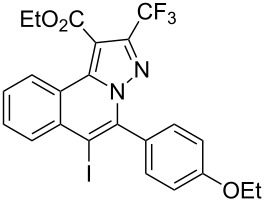 **4d**	88	12	**1j**	I_2_	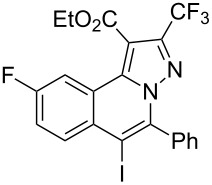 **4i**	50

^a^Reaction conditions: *N*’-(2-alkynylbenzylidene)hydrazide **1a** (0.3 mmol), I_2_ or ICl (1.3 equiv), solvent (3 mL), ethyl 4,4,4-trifluorobut-2-ynoate (**2**, 0.6 mmol, 2.0 equiv), base (1.5 equiv), 4 Å MS (75 mg), rt, overnight. ^b^Isolated yields based on N’-(2-alkynylbenzylidene)hydrazides **1**.

## Conclusion

In conclusion, we have developed an efficient one-pot tandem cyclization/[3 + 2] cycloaddition reaction of *N*’-(2-alkynylbenzylidene)hydrazides with ethyl 4,4,4-trifluorobut-2-ynoate under silver triflate-catalyzed or electrophiles-mediated conditions. Highly functionalized pyrazolo[5,1-*a*]isoquinolines can be synthesized in moderate to excellent yield by this developed method.

## Experimental

### General

All reactions were performed in test tubes under N_2_-atmosphere. Flash column chromatography was performed with silica gel (200–300 mesh). Analytical thin-layer chromatography was performed on glass plates pre-coated with 0.25 mm 230–400 mesh silica gel impregnated with a fluorescent indicator (254 nm). Thin-layer chromatography plates were visualized by exposure to ultraviolet light. Organic solutions were concentrated on rotary evaporators at 25–35 °C. Commercial reagents and solvents were used as received. ^1^H and ^13^C NMR spectra were recorded on a Bruker AV 400 at 400 MHz (^1^H) and 100 MHz (^13^C) at ambient temperature. Chemical shifts are reported in parts per million (ppm) on the delta scale (δ) and referenced to tetramethylsilane (0 ppm). HRMS analyses were performed in ESI mode on a Bruker mass spectrometer.

General procedure for the silver triflate-catalyzed one-pot tandem reaction of *N*’-(2-alkynylbenzylidene)hydrazide **1** with ethyl 4,4,4-trifluorobut-2-ynoate **2**: A mixture of *N*’-(2-alkynylbenzylidene)hydrazide **1** (0.30 mmol, 1.0 equiv) and silver triflate (5 mol %) in anhydrous dichloromethane (3.0 mL) was stirred at room temperature overnight. Then a solution of ethyl 4,4,4-trifluorobut-2-ynoate (**2**, 0.60 mmol, 2.0 equiv) in dichloroethane (2.0 mL), 4 Å MS (75 mg) and CsF (0.45 mmol, 1.5 equiv) were added and stirred for another 3 h. After completion of the reaction as indicated by TLC, the reaction mixture was purified by flash column chromatography on silica gel to provide the corresponding product **3**.

General procedure for the electrophile-mediated one-pot tandem reaction of *N*’-(2-alkynylbenzylidene)hydrazide **1** with ethyl 4,4,4-trifluorobut-2-ynoate (**2**): A mixture of *N*’-(2-alkynylbenzylidene)hydrazide **1** (0.30 mmol, 1.0 equiv) and electrophiles (I_2_ or ICl) (0.36 mmol, 1.2 equiv) in anhydrous dichloromethane (3.0 mL) was stirred at room temperature overnight. Then a solution of ethyl 4,4,4-trifluorobut-2-ynoate (**2**, 0.60 mmol, 2.0 equiv) in dichloroethane (2.0 mL), 4 Å MS (75 mg) and CsF (0.45 mmol, 1.5 equiv) were added and stirred for another 3 h. After completion of the reaction as indicated by TLC, the reaction mixture was purified by flash column chromatography on silica gel to provide the corresponding product **4**. For details, see Supporting Information File 1.

## Supporting Information

File 1 Characterization data and NMR spectra.

File 2 X-ray data for compound **3b**.

File 3 X-ray data for compound **4h**.
