# Supplementary material for: Synthesis of 2-trifluoromethylpyrazolo[5,1-a]isoquinolines via silver triflate-catalyzed or electrophile-mediated one-pot tandem reaction
Source: Beilstein J Org Chem. 2014 Sep 30;10:2286–92. doi: 10.3762/bjoc.10.238 (PMC4187034; doi:10.3762/bjoc.10.238)
Supplement: File 1 — Characterization data and NMR spectra. [file Beilstein_J_Org_Chem-10-2286-s001.pdf]

Supporting Information

for

Synthesis of 2-trifluoromethylpyrazolo[5,1-*a*]isoquinolines via silver triflate-catalyzed or electrophile-mediated one-pot tandem reaction

Meiling Liu[1], Puying Luo[* 2], Yingjun Lai[1], Tangtao Yang[1] and Qiuping Ding[* § 1]

Address: [1]Key Laboratory of Small Functional Organic Molecule, Ministry of Education and Jiangxi's Key Laboratory of green chemistry, Jiangxi Normal University, Nanchang, Jiangxi 330022, P. R. China and [2]Department of Obstetrics and Gynecology, Jiangxi Provincial people's Hospital, Nanchang, Jiangxi 330006, P. R. China.

Email: Q. Ding - dingqiuping@jxnu.edu.cn; P. Luo - luopuying1979@gmail.com

*Corresponding author

[§]Tel.: +86 791 88120380; fax: +86 791 88120380

Characterization data and NMR spectra

1. General procedure and characterization data for compounds **3** and **4** S2–S13

2. NMR spectra of all compounds. S14–S32

General procedure for the silver triflate-catalyzed one-pot tandem reaction of N'-(2-alkynylbenzylidene)-hydrazide **1** with ethyl 4,4,4-trifluorobut-2-ynoate **2** : A mixture of N'-(2-alkynylbenzylidene)hydrazide **1** (0.30 mmol, 1.0 equiv.) and silver triflate (5 mol%) in anhydrous dichloromethane (3.0 mL) was stirred at room temperature for overnight. Then a solution of ethyl 4,4,4-trifluorobut-2-ynoate (0.60 mmol, 2.0 equiv.) in dichloroethane (2.0 mL), 4A MS (75 mg) and CsF (0.45 mmol, 1.5 equiv.) were added, stirred for another 3 h. After completion of the reaction as indicated by TLC, the reaction mixture was purified by flash column chromatography on silica gel to provide the corresponding product **3**.

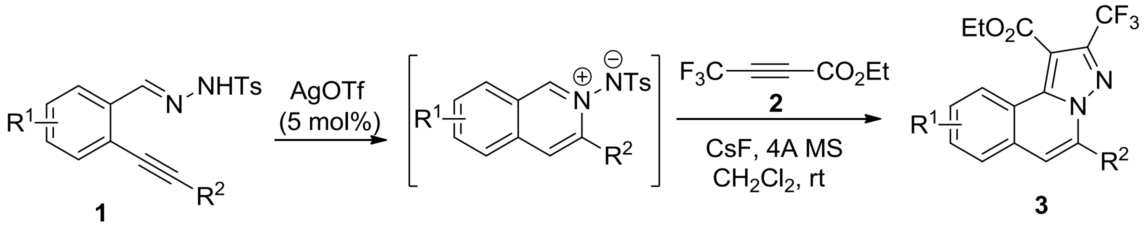

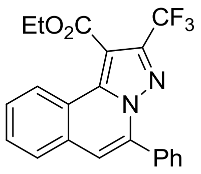

Ethyl 5-phenyl-2-(trifluoromethyl)pyrazolo[5,1-a]isoquinoline-1-carboxylate (**3a**) Isolated (Rf = 0.7, EtOAc–petroleum ether = 1:10) as a colorless solid (105 mg, 91% yield), mp: 147-148 ^oC; ^1H NMR (400 MHz, $CDCl_3$) δ 1.46 (t, *J* = 7.2 Hz, 3H), 4.49 (q, *J* = 7.2 Hz, 2H), 7.25 (s, 1H), 7.46-7.54 (m, 3H), 7.57-7.64 (m, 2H), 7.71-7.76 (m, 1H), 7.80-7.85 (m, 2H), 9.17 (d, *J* = 9.2 Hz, 1H); ^{13}C NMR (100 MHz, $CDCl_3$) δ 13.9, 61.7, 106.3, 116.6, 121.0 (q, $^1J_{C-F}$ = 269.0 Hz), 123.2, 126.3, 127.5, 128.1, 128.4, 129.7, 129.8, 130.0, 130.6, 132.4, 137.9, 140.1, 143.1 (q, $^2J_{C-F}$ = 37.0 Hz), 163.4; HRMS calcd. For $C_{21}H_{16}F_3N_2O_2$ $[M+H]^+$: 385.1164, Found: 385.1167.

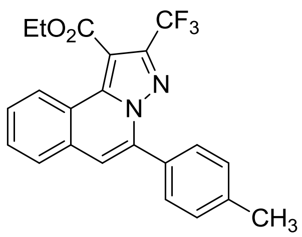

Ethyl 5-(p-tolyl)-2-(trifluoromethyl)pyrazolo[5,1-a]isoquinoline-1-carboxylate (**3b**) Isolated (Rf = 0.7, EtOAc–petroleum ether = 1:10) as a colorless solid (102 mg, 86% yield), mp: 133-134 ^{o}C; ^{1}H NMR (400 MHz, $CDCl_3$) δ 1.46 (t, J = 7.2 Hz, 3H), 2.43(s, 3H), 4.49 (q, J = 7.2 Hz, 2H), 7.23 (s, 1H), 7.30 (d, J = 8.0 Hz, 2H), 7.57-7.63 (m, 2H), 7.72 (d, J = 8.0 Hz, 3H), 9.16 (d, J = 8.8 Hz, 1H); ^{13}C NMR (100 MHz, $CDCl_3$) δ 13.9, 21.5, 61.7, 106.2, 116.2, 121.1 (q, $^{1}J_{C\text{-}F}$ = 269.0 Hz), 123.1, 126.3, 127.4, 127.9, 129.1, 129.4, 129.6, 129.9, 130.7, 138.0, 139.9, 140.1, 143.0 (q, $^{2}J_{C\text{-}F}$ = 37.0 Hz), 163.4; HRMS calcd. For. $C_{22}H_{17}F_3N_2NaO_2$ $[M+Na]^+$: 421.1140, Found: 421.1155.

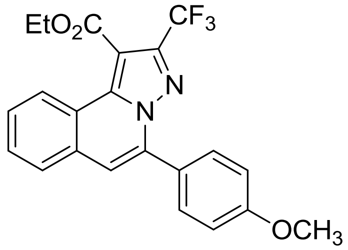

Ethyl 5-(4-methoxyphenyl)-2-(trifluoromethyl)pyrazolo[5,1-a]isoquinoline-1-carboxylate (**3c**) Isolated (Rf = 0.7, EtOAc–petroleum ether = 1:10) as a colorless solid (93.1 mg, 75% yield); ^{1}H NMR (400 MHz, $CDCl_3$) δ 1.38 (t, J = 7.2 Hz, 3H), 3.78 (s, 3H), 4.49 (q, J = 7.2 Hz, 2H), 6.93 (d, J = 7.2 Hz, 2H), 7.12 (s, 1H), 7.48-7.54 (m, 2H), 7.62 (d, J = 7.2 Hz, 2H), 7.70 (d, J = 8.8 Hz, 2H), 9.07 (d, J = 7.2 Hz, 1H); ^{13}C NMR (100 MHz, $CDCl_3$) δ 13.9, 55.4, 61.7, 106.2, 113.8, 115.8, 121.1 (q, $^{1}J_{C\text{-}F}$ = 269.0 Hz), 123.0, 124.6, 126.2, 127.3, 127.8, 129.8, 130.7, 131.1, 137.7, 140.1, 142.9

(q, $^{2}J_{C\text{-}F}$ = 37.0 Hz), 160.7, 163.4; HRMS calcd. For $C_{22}H_{17}F_3KN_2O_3$ $[M+K]^+$: 453.0823, Found: 453.0850.

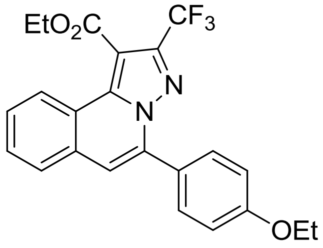

Ethyl 5-(4-ethoxyphenyl)-2-(trifluoromethyl)pyrazolo[5,1-a]isoquinoline-1-carboxylate (**3d**) Isolated (Rf = 0.7, EtOAc–petroleum ether = 1:10) as a colorless solid (114 mg, 89% yield), mp: 137-138 °C; ^{1}H NMR (400 MHz, $CDCl_3$) δ 1.45 (t, *J* = 6.8 Hz, 3H), 1.47 (t, *J* = 6.8 Hz, 3H), 4.12 (q, *J* = 6.8 Hz, 2H), 4.50 (q, *J* = 7.2 Hz, 2H), 7.03 (d, *J* = 8.0 Hz, 2H), 7.26 (s, 1H), 7.60-7.68 (m, 2H), 7.77 (d, *J* = 8.4 Hz, 1H), 7.80 (d, *J* = 8.4 Hz, 2H), 9.18 (d, *J* = 8.8 Hz, 1H); ^{13}C NMR (100 MHz, $CDCl_3$) δ 13.9, 14.8, 61.7, 63.6, 106.2, 114.3, 115.7, 121.1 (q, $^{1}J_{C\text{-}F}$ = 268.0 Hz), 123.0, 124.4, 126.2, 127.3, 127.7, 129.8, 130.8, 131.1, 137.8, 140.1, 143.0 (q, $^{2}J_{C\text{-}F}$ = 37.0 Hz), 160.1, 163.4; HRMS calcd. For $C_{23}H_{20}F_3N_2O_3$ $[M+H]^+$: 429.1426, Found: 429.1434.

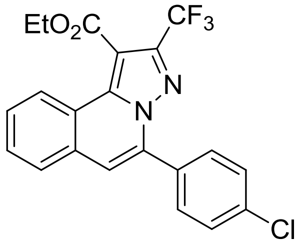

Ethyl 5-(4-chlorophenyl)-2-(trifluoromethyl)pyrazolo[5,1-a]isoquinoline-1-carboxylate (**3e**) Isolated (Rf = 0.7, EtOAc–petroleum ether = 1:10) as a colorless solid (106 mg, 85% yield), mp: 174-175 °C; ^{1}H NMR (400 MHz, $CDCl_3$) δ 1.46 (t, *J* = 6.8 Hz, 3H), 4.50 (q, *J* = 6.8 Hz, 2H), 7.29 (s, 1H), 7.50 (d, *J* = 8.0 Hz, 2H), 7.65-7.73 (m, 2H), 7.80 (d, *J* = 8.8 Hz, 3H), 9.21 (d, *J* = 6.8 Hz, 1H); ^{13}C NMR (100 MHz, $CDCl_3$)

δ 13.8, 61.7, 106.5, 116.6, 120.8 (q, ${}^{1}J_{C\text{-}F}$ = 268.0 Hz), 123.4, 126.4, 127.5, 128.4, 128.7, 130.1, 130.4, 130.8, 131.0, 135.8, 136.8, 140.1, 143.1 (q, ${}^{2}J_{C\text{-}F}$ = 37.0 Hz), 163.3; HRMS calcd. For $C_{21}H_{14}ClF_3N_2NaO_2$ $[M+Na]^+$: 441.0594, Found: 441.0599.

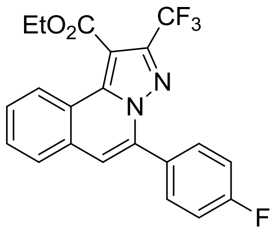

Ethyl 5-(4-fluorophenyl)-2-(trifluoromethyl)pyrazolo[5,1-a]isoquinoline-1-carboxylate (**3f**) Isolated (Rf = 0.7, EtOAc–petroleum ether = 1:5) as a yellow solid (96 mg, 80% yield); ^{1}H NMR (400 MHz, $CDCl_3$) δ 1.46 (t, J = 7.2 Hz, 3H), 4.50 (q, J = 7.2 Hz, 2H), 7.21 (t, J = 8.8 Hz, 2H), 7.26 (s, 1H), 7.63-7.68 (m, 2H), 7.77 (d, J = 8.0 Hz, 1H), 7.81-7.88 (m, 2H), 9.19 (d, J = 7.6 Hz, 1H); ^{13}C NMR (100 MHz, $CDCl_3$) δ 13.9, 61.8, 106.4, 115.6 (d, ${}^{2}J_{C\text{-}F}$ = 22.0 Hz), 116.5, 121.0 (q, ${}^{1}J_{C\text{-}F}$ = 269.0 Hz), 123.3, 126.3, 127.5, 128.2, 128.4, 130.0, 130.5, 131.7 (d, ${}^{3}J_{C\text{-}F}$ = 8.0 Hz), 136.9, 140.1, 143.1 (q, ${}^{2}J_{C\text{-}F}$ = 37.0 Hz), 163.3, 163.5 (d, ${}^{1}J_{C\text{-}F}$ = 249.0 Hz); HRMS calcd. for $C_{21}H_{15}F_4N_2O_2$ $[M+H]^+$: 403.1070, Found: 403.1064.

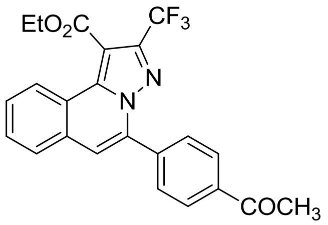

Ethyl 5-(4-acetylphenyl)-2-(trifluoromethyl)pyrazolo[5,1-a]isoquinoline-1-carboxylate (**3g**) Isolated (Rf = 0.7, EtOAc–petroleum ether = 1:10) as a colorless solid (111 mg, 87% yield), mp: 167-168 ^{o}C; ^{1}H NMR (400 MHz, $CDCl_3$) δ 1.46 (t, J = 6.8 Hz,

3H), 2.67 (s, 3H), 4.50 (q, J = 7.2 Hz, 2H), 7.33 (s, 1H), 7.63-7.69 (m, 2H), 7.75-7.80 (m, 1H), 7.94 (d, J = 8.0 Hz, 2H), 8.08 (d, J = 7.6 Hz, 2H), 9.19 (d, J = 8.0 Hz, 1H); ^{13}C NMR (100 MHz, CDCl$_3$) δ 13.8, 26.7, 61.7, 106.4, 117.1, 120.8 (q, $^1J_{C\text{-}F}$ = 269.0 Hz), 123.4, 126.3, 127.6, 128.2, 128.5, 129.8, 130.0, 130.2, 136.6, 137.6, 140.0, 143.0 (q, $^2J_{C\text{-}F}$ = 37.0 Hz), 163.1, 197.4; HRMS calcd. for $C_{23}H_{18}F_3N_2O_3$ [M+H]$^+$: 427.1270, Found: 427.1276.

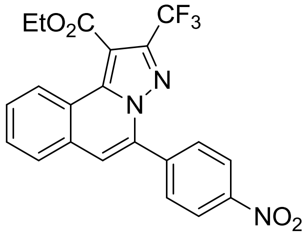

Ethyl 5-(4-nitrophenyl)-2-(trifluoromethyl)pyrazolo[5,1-a]isoquinoline-1-carboxylate (**3h**) Isolated (Rf = 0.7, EtOAc–petroleum ether = 1:5) as a pale yellow solid (60.5 mg, 47% yield), mp: 182-183 °C; ^{1}H NMR (400 MHz, CDCl$_3$) δ 1.46 (t, J = 7.2 Hz, 3H), 4.49 (q, J = 7.2 Hz, 2H), 7.27 (s, 1H), 7.49 (d, J = 8.4 Hz, 2H), 7.61-7.70 (m, 2H), 7.72-7.81 (m, 3H), 9.18 (d, J = 9.2 Hz, 1H); ^{13}C NMR (100 MHz, CDCl$_3$) δ 13.8, 61.7, 106.4, 116.6, 120.9 (q, $^1J_{C\text{-}F}$ = 269.0 Hz), 123.3, 126.3, 127.5, 128.3, 128.7, 130.0, 130.4, 130.7, 131.0, 135.8, 136.7, 140.1, 143.1 (q, $^2J_{C\text{-}F}$ = 37.0 Hz), 163.3; HRMS calcd. for $C_{21}H_{15}F_3N_3O_4$ [M+H]$^+$: 430.1015, Found: 430.1021.

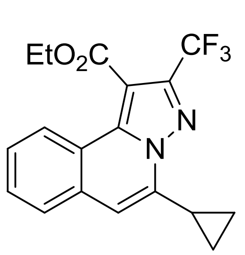

Ethyl 5-cyclopropyl-2-(trifluoromethyl)pyrazolo[5,1-a]isoquinoline-1-carboxylate (**3i**) Isolated (Rf = 0.7, EtOAc–petroleum ether = 1:10) as a colorless solid (94 mg, 90%

yield), mp: 75-76 °C; ^{1}H NMR (400 MHz, $CDCl_3$) δ 0.80-0.90 (m, 2H), 1.13 (d, *J* = 7.2 Hz, 2H), 1.37 (t, *J* = 6.4 Hz, 3H), 2.60-2.65 (m, 1H), 4.40 (d, *J* = 6.4 Hz, 2H), 6.73 (s, 1H), 7.35-7.54 (m, 3H), 9.05 (d, *J* = 7.2 Hz, 1H); ^{13}C NMR (100 MHz, $CDCl_3$) δ 7.6, 11.1, 13.9, 61.6, 106.2, 110.4, 121.2 (q, $^1J_{C\text{-}F}$ = 268.0 Hz), 122.5, 126.2, 126.7, 127.2, 129.6, 130.5, 139.5, 140.7, 143.0 (q, $^2J_{C\text{-}F}$ = 37.0 Hz), 163.4; HRMS calcd. For $C_{18}H_{15}F_3N_2NaO_2$ $[M+Na]^+$: 371.0983, Found: 371.0991.

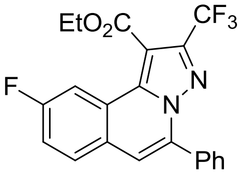

Ethyl 9-fluoro-5-phenyl-2-(trifluoromethyl)pyrazolo[5,1-a]isoquinoline-1-carboxylate (**3j**) Isolated (Rf = 0.6, EtOAc–petroleum ether = 1:10) as a colorless solid (53 mg, 44% yield), mp: 161-162 °C; ^{1}H NMR (400 MHz, $CDCl_3$) δ 1.46 (t, *J* = 7.2 Hz, 3H), 4.50 (q, *J* = 7.2 Hz, 2H), 7.30 (s, 1H), 7.44 (dt, *J* = 2.4, 8.4 Hz, 1H), 7.50-7.56 (m, 3H), 7.78-7.85 (m, 3H), 9.09 (dd, *J* = 2.0, 10.8 Hz, 1H); ^{13}C NMR (100 MHz, $CDCl_3$) δ 13.8, 61.8, 106.8, 112.2 (d, $^2J_{C\text{-}F}$ = 26.0 Hz), 115.8, 118.9 (d, $^2J_{C\text{-}F}$ = 26.0 Hz), 120.9 (d, $^1J_{C\text{-}F}$ = 268.0 Hz), 124.7 (d, $^3J_{C\text{-}F}$ = 11.0 Hz), 127.3, 128.4, 129.6 (d, $^3J_{C\text{-}F}$ = 9.0 Hz), 129.7, 129.8, 132.2, 137.5, 139.5, 143.4 (d, $^2J_{C\text{-}F}$ = 37.0 Hz), 161.7 (d, $^1J_{C\text{-}F}$ = 247.0 Hz), 163.0; HRMS calcd. for $C_{21}H_{15}F_4N_2O_2$ $[M+H]^+$: 403.1070, Found 403.1064.

General procedure for the electrophile-mediated one-pot tandem reaction of N'-(2-alkynylbenzylidene)-hydrazide **1** with ethyl 4,4,4-trifluorobut-2-ynoate **2** : A mixture of N'-(2-alkynylbenzylidene)hydrazide **1** (0.30 mmol, 1.0 equiv.) and

electrophiles (I_2 or ICl) (0.36 mmol, 1.2 equiv.) in anhydrous dichloromethane (3.0 mL) was stirred at room temperature for overnight. Then a solution of ethyl 4,4,4-trifluorobut-2-ynoate (0.60 mmol, 2.0 equiv.) in dichloroethane (2.0 mL), 4A MS (75 mg) and CsF (0.45 mmol, 1.5 equiv.) were added, stirred for another 3 h. After completion of the reaction as indicated by TLC, the reaction mixture was purified by flash column chromatography on silica gel to provide the corresponding product **4**.

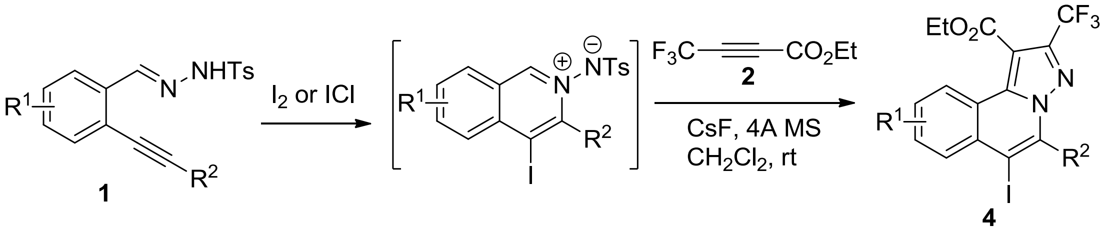

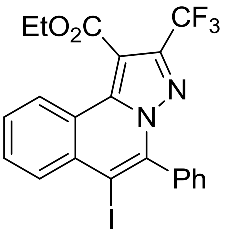

Ethyl 6-iodo-5-phenyl-2-(trifluoromethyl)pyrazolo[5,1-a]isoquinoline-1-carboxylate (**4a**) Isolated (Rf = 0.7, EtOAc–petroleum ether = 1:10) as a yellow solid (119 mg, 78% yield); ^{1}H NMR (400 MHz, $CDCl_3$) δ 1.44 (t, *J* = 7.2 Hz, 3H), 4.49 (q, *J* = 7.2 Hz, 2H), 7.38-7.45 (m, 2H), 7.51-7.61 (m, 3H), 7.63-7.74 (m, 2H), 8.24 (d, *J* = 8.0 Hz, 1H), 9.17 (d, *J* = 8.8 Hz, 1H); ^{13}C NMR (100 MHz, $CDCl_3$) δ 13.8, 61.9, 92.4, 106.2, 120.7 (q, $^1J_{C\text{-}F}$ = 270.0 Hz), 123.0, 126.5, 128.7, 129.1, 129.8, 130.3, 131.0, 131.4, 133.5, 136.8, 139.2, 140.9, 143.2 (q, $^2J_{C\text{-}F}$ = 38.0 Hz), 163.3; HRMS calcd. for $C_{21}H_{15}F_3IN_2O_2$ $[M+H]^+$: 511.0130, Found 511.0125.

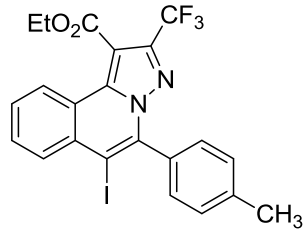

Ethyl 6-iodo-5-(p-tolyl)-2-(trifluoromethyl)pyrazolo[5,1-a]isoquinoline-1-carboxylate (**4b**) Isolated (Rf = 0.7, EtOAc–petroleum ether = 1:10) as a yellow solid (141 mg, 90% yield), mp: 165-166 °C; ^{1}H NMR (400 MHz, $CDCl_3$) δ 1.44 (t, J = 7.2 Hz, 3H), 2.48 (s, 3H), 4.48 (q, J = 7.2 Hz, 2H), 7.30 (d, J = 7.6 Hz, 2H), 7.37 (d, J = 7.6 Hz, 2H), 7.63-7.71 (m, 2H), 8.22 (d, J = 8.0 Hz, 1H), 9.14 (d, J = 8.0 Hz, 1H); ^{13}C NMR (100 MHz, $CDCl_3$) δ 13.7, 21.6, 61.7, 92.4, 106.1, 120.6 (q, $^1J_{C\text{-}F}$ = 269.0 Hz), 122.8, 126.3, 126.9, 128.8, 129.3, 130.0, 130.8, 131.3, 133.4, 133.7, 139.1, 139.7, 140.9, 143.0 (q, $^2J_{C\text{-}F}$ = 37.0 Hz), 163.2; HRMS calcd. for $C_{22}H_{16}F_3IN_2NaO_2$ $[M+Na]^+$: 547.0126, found 547.0101.

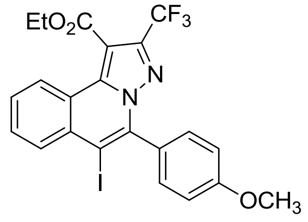

Ethyl 6-iodo-5-(4-methoxyphenyl)-2-(trifluoromethyl)pyrazolo[5,1-a]isoquinoline-1-carboxylate (**4c**) Isolated (Rf = 0.5, EtOAc–petroleum ether = 1:20) as a yellow solid (130 mg, 80% yield), mp: 154-155 °C; ^{1}H NMR (400 MHz, $CDCl_3$) δ 1.44 (t, J = 7.2 Hz, 3H), 3.92 (s, 3H), 4.49 (q, J = 7.2 Hz, 2H), 7.09 (d, J = 8.0 Hz, 2H), 7.36 (d, J = 8.4 Hz, 2H), 7.68-7.76 (m, 2H), 8.27 (d, J = 8.0 Hz, 1H), 9.17 (d, J = 8.0 Hz, 1H); ^{13}C NMR (100 MHz, $CDCl_3$) δ 13.8, 55.3, 61.9, 92.9, 106.2, 114.0, 119.7 (q, $^1J_{C\text{-}F}$ = 269.0 Hz), 123.0, 126.4, 129.0, 129.1, 131.0, 131.6, 131.8, 133.6, 139.2, 140.9, 143.0 (q,

$^{2}J_{C\text{-}F}$ = 37.0 Hz), 160.5, 163.4; HRMS calcd. for $C_{22}H_{17}F_3IN_2O_3$ $[M+H]^+$: 541.0236, Found 541.0230.

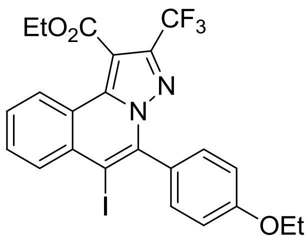

Ethyl 5-(4-ethoxyphenyl)-6-iodo-2-(trifluoromethyl)pyrazolo[5,1-a]isoquinoline-1-carboxylate (**4d**) Isolated (Rf = 0.7, EtOAc–petroleum ether = 1:10) as a yellow solid (145 mg, 88% yield); ^{1}H NMR (400 MHz, $CDCl_3$) δ 1.45 (t, *J* = 6.8 Hz, 3H), 1.47 (t, *J* = 6.8 Hz, 3H), 4.13 (q, *J* = 7.2 Hz, 2H), 4.48 (d, *J* = 7.2 Hz, 2H), 7.06 (d, *J* = 8.4 Hz, 2H), 7.34 (d, *J* = 8.4 Hz, 2H), 7.64-7.69 (m, 2H), 8.20 (d, *J* = 8.0 Hz,1H), 9.14 (d, *J* = 8.0 Hz,1H); ^{13}C NMR (100 MHz, $CDCl_3$) δ 12.8, 13.8, 60.7, 62.4, 91.8, 105.1, 113.3, 119.7 (q, $^{1}J_{C\text{-}F}$ = 269.0 Hz), 121.8, 125.3, 127.7, 127.8, 129.8, 130.4, 130.8, 132.4, 138.1, 139.8, 142.0 (q, $^{1}J_{C\text{-}F}$ = 37.0 Hz), 158.8, 162.2; HRMS calcd. for $C_{23}H_{19}F_3IN_2O_3$ $[M+H]^+$: 555.0392, Found 555.0387 .

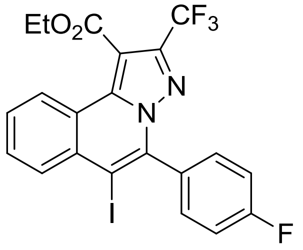

Ethyl 5-(4-fluorophenyl)-6-iodo-2-(trifluoromethyl)pyrazolo[5,1-a]isoquinoline-1-carboxylate (**4e**) Isolated (Rf = 0.7, EtOAc–petroleum ether = 1:10) as a yellow solid (97 mg, 62% yield), mp: 197-198 °C; ^{1}H NMR (400 MHz, $CDCl_3$) δ 1.44 (t, *J* = 7.2 Hz, 3H), 4.48 (q, *J* = 7.2 Hz, 2H), 7.26 (t, *J* = 8.4 Hz, 2H), 7.36-7.45 (m, 2H), 7.64-7.72 (m, 2H), 8.20 (d, *J* = 7.6 Hz, 1H), 9.15 (d, *J* = 8.0 Hz, 1H); ^{13}C NMR (100

MHz, $CDCl_3$) δ 13.8, 61.9, 92.8, 106.3, 116.0 (d, $^2J_{C\text{-}F}$ = 22.0 Hz), 120.7 (q, $^1J_{C\text{-}F}$ = 269.0 Hz), 123.0, 126.5, 129.2, 131.1, 131.3, 132.5 (d, $^3J_{C\text{-}F}$ = 9.0 Hz), 132.7, 133.5, 139.2, 139.9, 143.3 (q, $^2J_{C\text{-}F}$ = 37.0 Hz), 163.2, 163.4 (d, $^1J_{C\text{-}F}$ = 249.0 Hz); HRMS calcd. for $C_{21}H_{14}F_4IN_2O_2$ $[M+H]^+$: 529.0036, Found 529.0031.

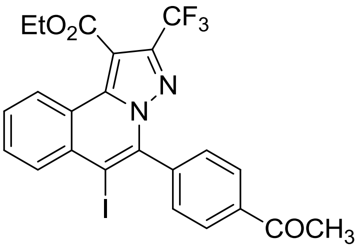

Ethyl 5-(4-acetylphenyl)-6-iodo-2-(trifluoromethyl)pyrazolo[5,1-a]isoquinoline-1-carboxylate (**4f**) Isolated (Rf = 0.7, EtOAc–petroleum ether = 1:10) as a yellow solid (139 mg, 84% yield); ^1H NMR (400 MHz, $CDCl_3$) δ 1.35 (t, *J* = 7.2 Hz, 3H), 2.62 (s, 3H), 4.40 (q, *J* = 7.2 Hz, 2H), 7.45 (d, *J* = 8.0 Hz, 2H), 7.60-7.70 (m, 2H), 8.08 (d, *J* = 8.0 Hz, 2H), 8.16 (d, *J* = 8.0 Hz, 1H), 9.10 (d, *J* = 8.0 Hz, 1H); ^{13}C NMR (100 MHz, $CDCl_3$) δ 13.8, 26.8, 61.9, 92.0, 106.4, 120.6 (q, $^1J_{C\text{-}F}$ = 269.0 Hz), 123.1, 126.6, 128.7, 129.4, 130.8, 131.2, 132.7, 133.5, 137.8, 139.2, 139.8, 141.0, 143.4 (q, $^2J_{C\text{-}F}$ = 38.0 Hz), 163.1, 197.5; HRMS calcd. for $C_{23}H_{17}F_3IN_2O_3$ $[M+H]^+$: 553.0236, Found 553.0230.

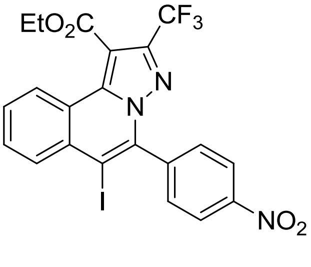

Ethyl 6-iodo-5-(4-nitrophenyl)-2-(trifluoromethyl)pyrazolo[5,1-a]isoquinoline-1-carboxylate (**4g**) Isolated (Rf = 0.7, EtOAc–petroleum ether = 1:10) as a colorless solid (83.2 mg, 50% yield); ^1H NMR (400 MHz, $CDCl_3$) δ 1.45 (t, *J* = 7.2 Hz, 3H),

4.49 (q, J = 7.2 Hz, 2H), 7.37 (d, J = 8.4 Hz, 2H), 7.56 (d, J = 8.0 Hz, 2H), 7.70-7.79 (m, 2H), 8.27 (d, J = 8.0 Hz, 1H), 9.19 (d, J = 8.0 Hz, 1H); ^{13}C NMR (100 MHz, $CDCl_3$) δ 12.8, 60.9, 91.5, 105.3, 115.5, 119.6 (q, $^1J_{C-F}$ = 269.0 Hz), 122.1, 125.5, 128.1, 128.6, 130.2, 130.8, 132.5, 133.9, 134.8, 138.1, 138.7, 142.3 (q, $^2J_{C-F}$ = 37.0 Hz), 162.2; HRMS calcd. for $C_{21}H_{14}F_3IN_3O_4$ $[M+H]^+$: 555.9981, Found: 555.9989.

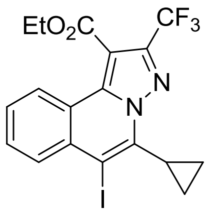

Ethyl 5-cyclopropyl-6-iodo-2-(trifluoromethyl)pyrazolo[5,1-a]isoquinoline-1-carboxylate (**4h**) Isolated (Rf = 0.7, EtOAc–petroleum ether = 1:10) as a colorless solid (122 mg, 85% yield), mp: 102-103 ^{o}C; ^{1}H NMR (400 MHz, $CDCl_3$) δ 1.16 (t, J = 5.6 Hz, 2H), 1.42-1.50 (m, 5H), 2.28-2.32 (m, 1H), 4.49 (q, J = 7.2 Hz, 2H), 7.58 (t, J = 7.2 Hz, 1H), 7.65 (t, J = 7.6 Hz, 1H), 8.18 (d, J = 8.0 Hz, 1H), 9.05 (d, J = 8.2 Hz, 1H); ^{13}C NMR (100 MHz, $CDCl_3$) δ 12.5, 13.8, 18.3, 61.7, 93.9, 105.9, 120.9 (q, $^1J_{C-F}$ = 268.0 Hz), 122.2, 126.1, 128.4, 130.6, 131.5, 132.5, 139.1, 140.2, 142.5 (q, $^2J_{C-F}$ = 37.0 Hz), 163.3; HRMS calcd. for $C_{18}H_{15}F_3IN_2O_2$ $[M+H]^+$: 475.0130, Found: 475.0125.

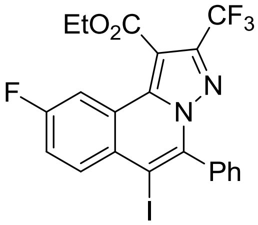

Ethyl 9-fluoro-6-iodo-5-phenyl-2-(trifluoromethyl)pyrazolo[5,1-a]isoquinoline-1-carboxylate (**4i**) Isolated (Rf = 0.7, EtOAc–petroleum ether = 1:10) as a colorless

solid (79 mg, 50% yield); ^{1}H NMR (400 MHz, $CDCl_3$) δ 1.44 (t, *J* = 7.2 Hz, 3H), 4.48 (q, *J* = 7.2 Hz, 2H), 7.37-7.48 (m, 3H), 7.52-7.65 (m, 3H), 8.27 (dd, *J* = 5.2, 8.8 Hz, 1H), 9.11 (d, *J* = 10.4 Hz, 1H); ^{13}C NMR (100 MHz, $CDCl_3$) δ 13.8, 62.0, 91.3, 106.6, 112.2 (d, $^{2}J_{C-F}$ = 26.0 Hz), 119.5 (d, $^{2}J_{C-F}$ = 23.0 Hz), 120.6 (q, $^{1}J_{C-F}$ = 269.0 Hz), 124.1 (d, $^{3}J_{C-F}$ = 10.0 Hz), 128.2, 128.7, 129.9, 130.3, 136.1 (d, $^{3}J_{C-F}$ = 8.0 Hz), 136.6, 138.7, 140.4, 143.5 (q, $^{2}J_{C-F}$ = 38.0 Hz), 162.2 (d, $^{1}J_{C-F}$ = 248.0 Hz), 162.9; HRMS calcd. for $C_{21}H_{14}F_4IN_2O_2$ $[M+H]^+$: 529.0036, Found: 529.0035.

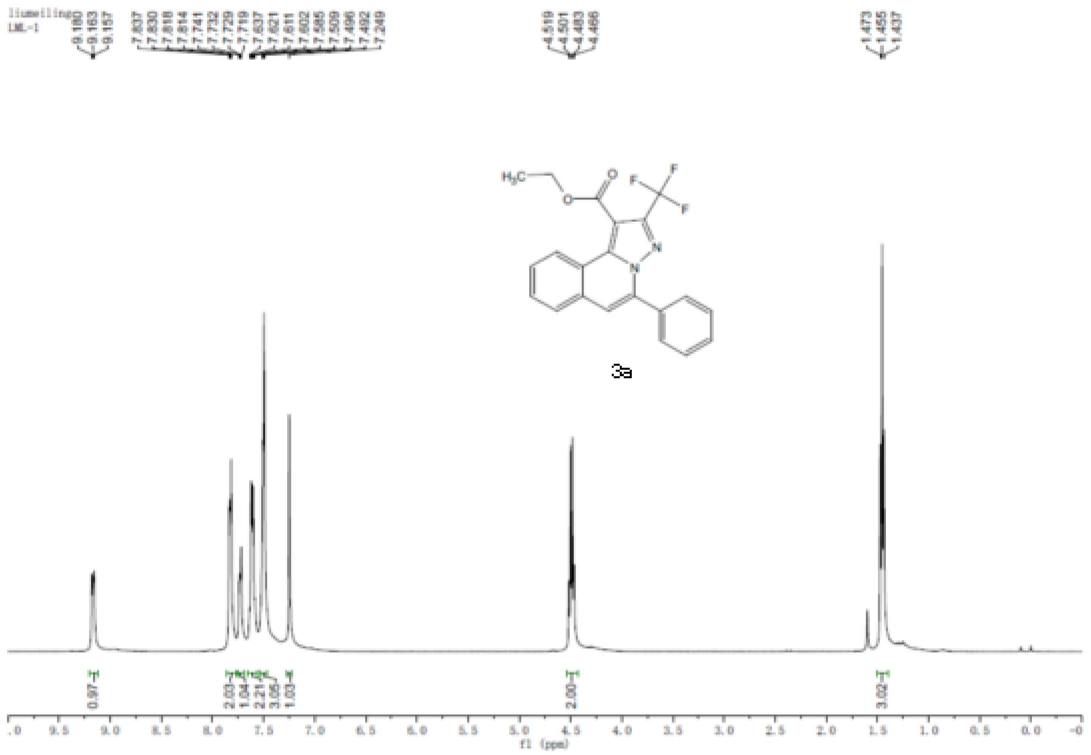

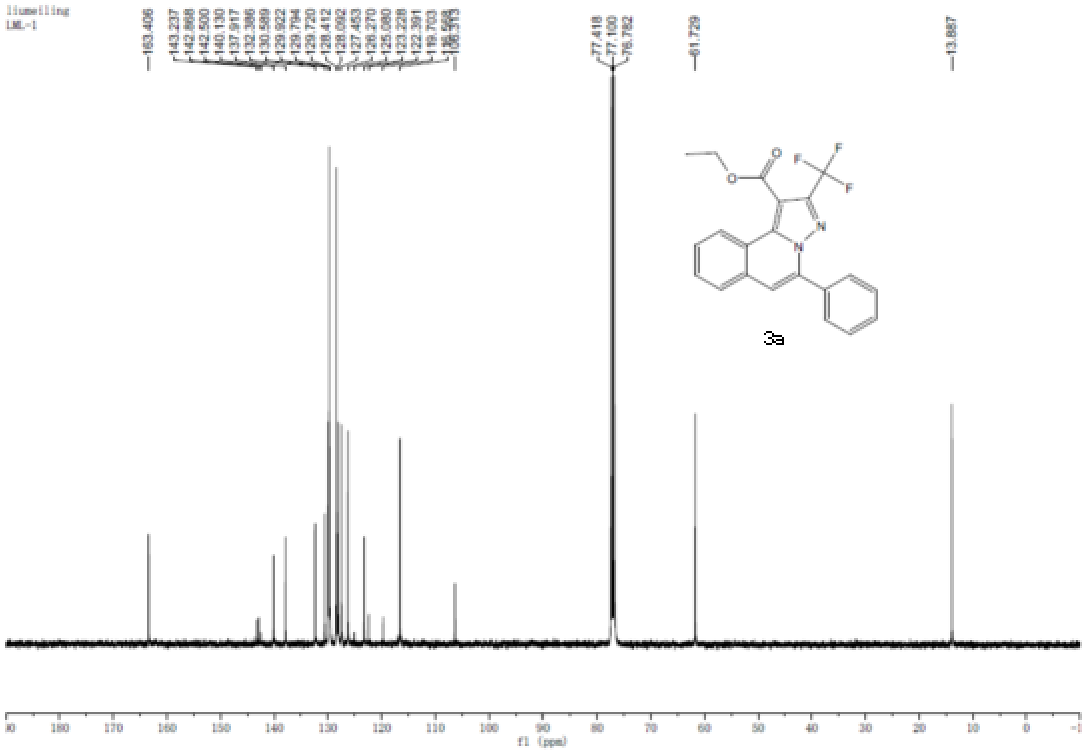

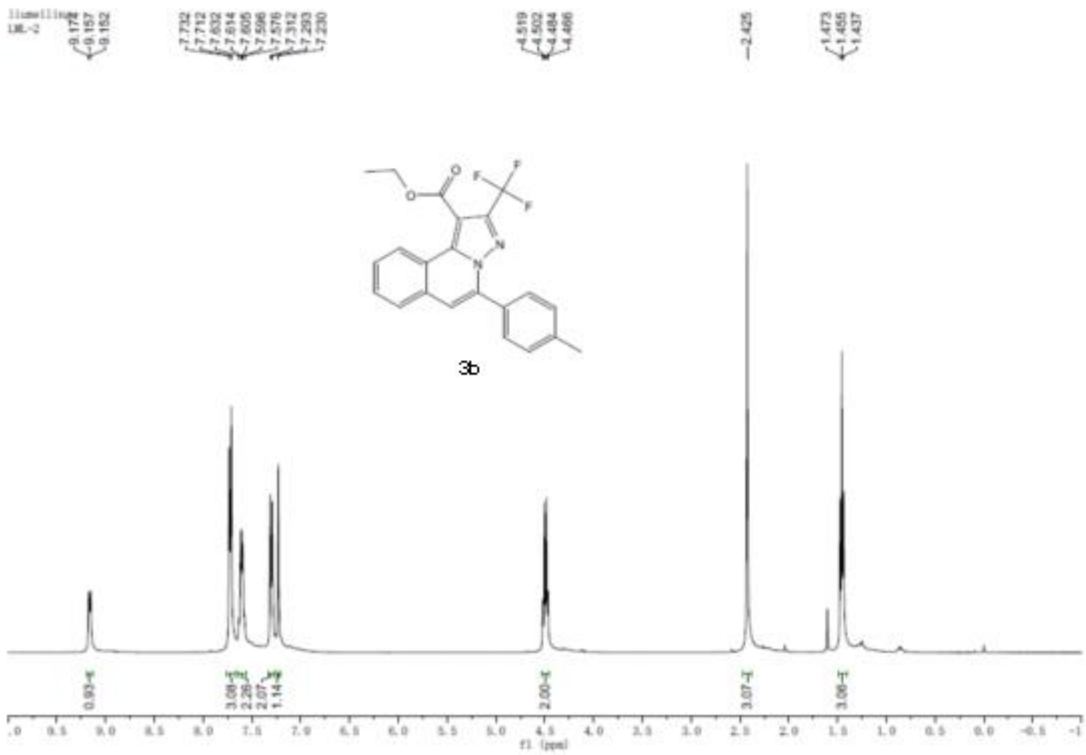

liumeiling
LML-2
9.172
9.157
9.152
7.732
7.712
7.632
7.614
7.605
7.596
7.576
7.312
7.293
7.230
4.519
4.502
4.484
4.466
2.425
1.473
1.455
1.437
3b
0.93
3.08
2.26
2.07
1.14
2.00
3.07
3.06
f1 (ppm)

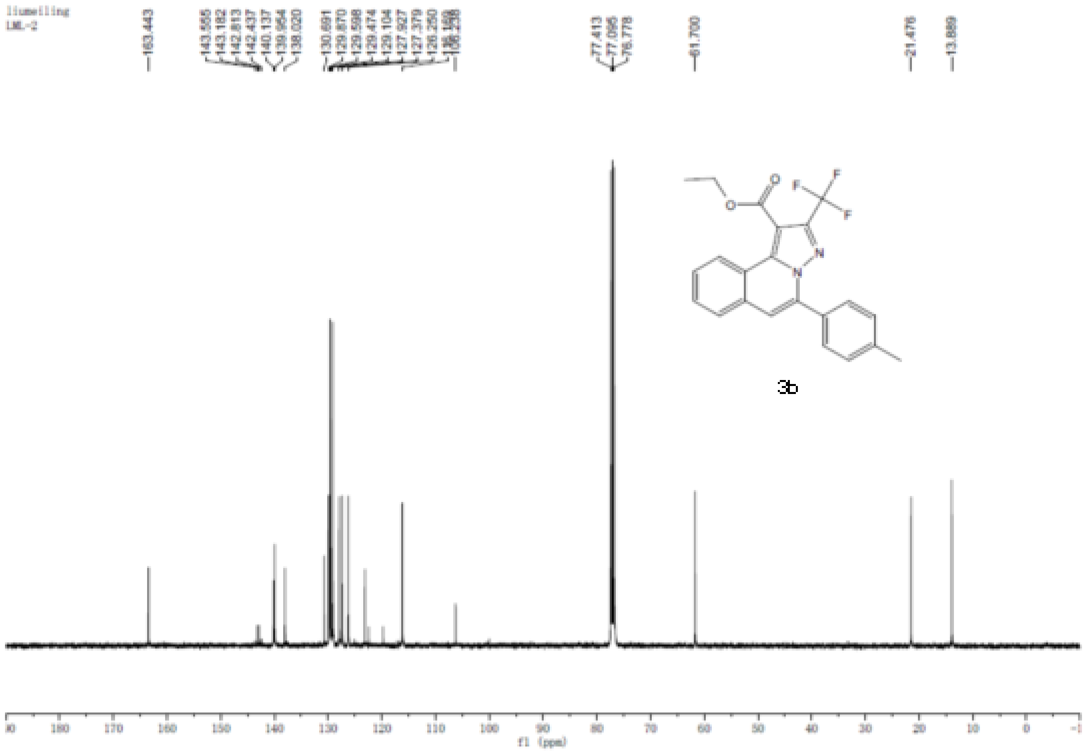

liumeiling
LML-2
163.443
143.555
143.182
142.813
142.437
140.137
139.954
138.020
130.691
129.870
129.598
129.474
129.104
127.927
127.379
126.250
77.413
77.095
76.778
61.700
21.476
13.889
3b
f1 (ppm)

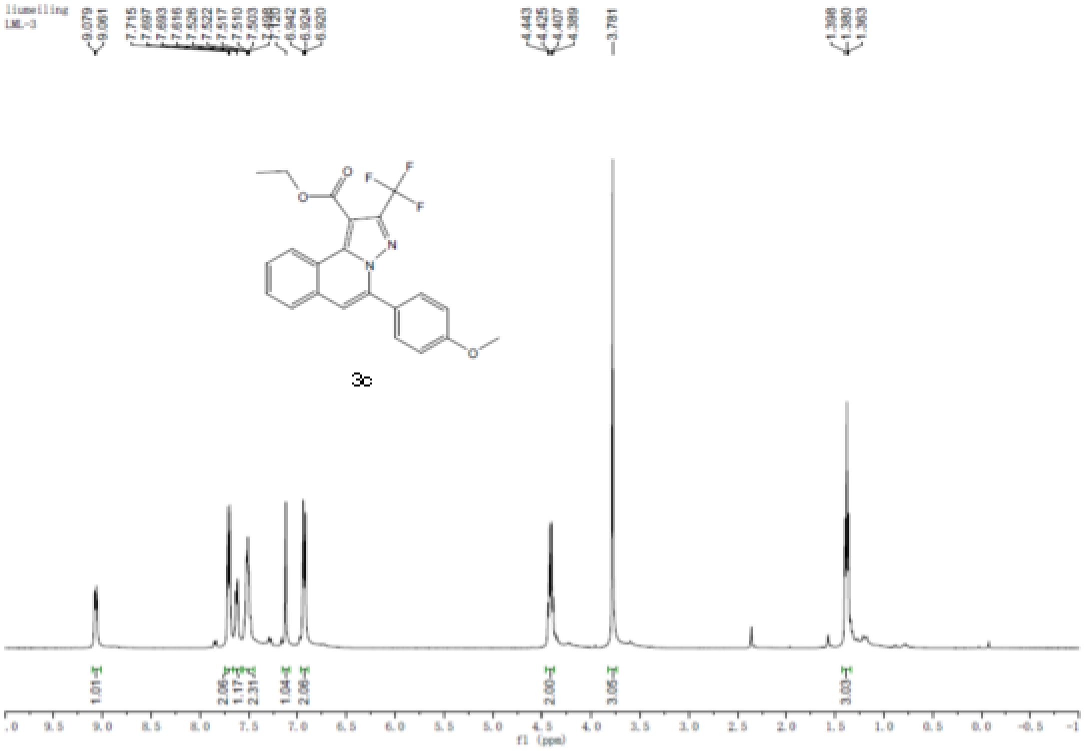
liumeiling
LML-3
9.079
9.061
7.715
7.697
7.693
7.616
7.526
7.522
7.517
7.510
7.503
6.942
6.924
6.920
4.443
4.425
4.407
4.389
3.781
1.398
1.380
1.363
3c
1.01
2.06
1.17
2.31
1.04
2.06
2.00
3.05
3.03
f1 (ppm)

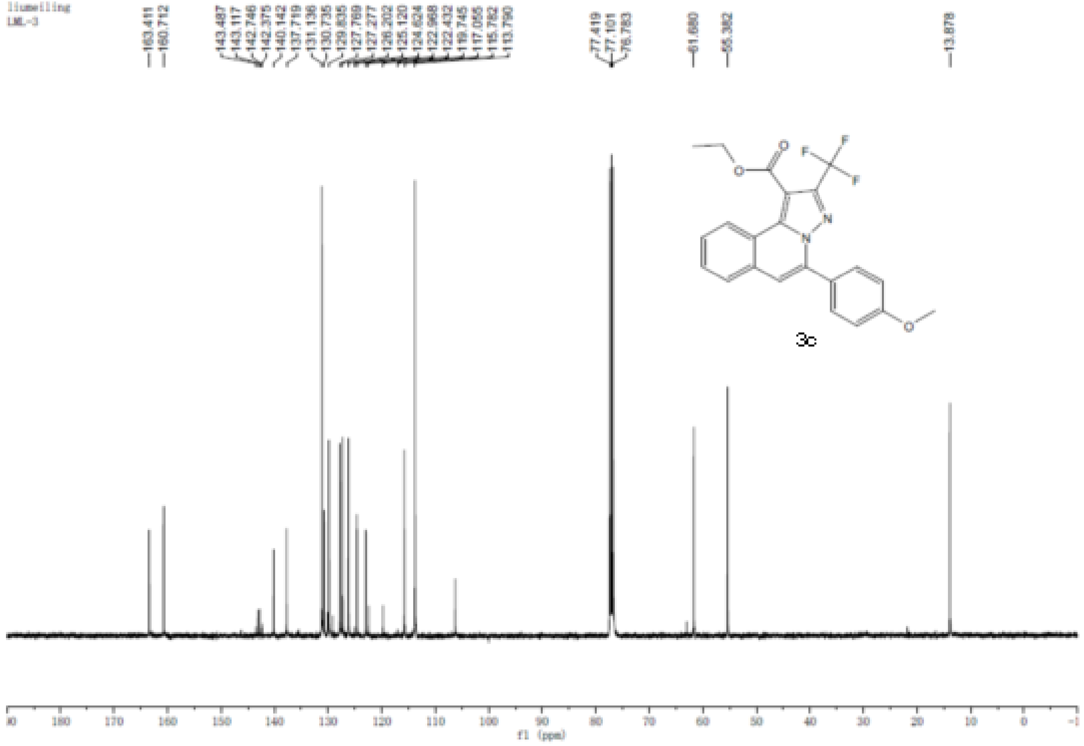
liumeiling
LML-3
163.411
160.712
143.487
143.117
142.746
142.375
140.142
137.719
131.136
130.735
129.835
127.769
127.277
126.202
125.120
124.624
122.968
122.432
119.745
117.055
115.782
113.790
77.419
77.101
76.783
61.680
55.382
13.878
3c
f1 (ppm)

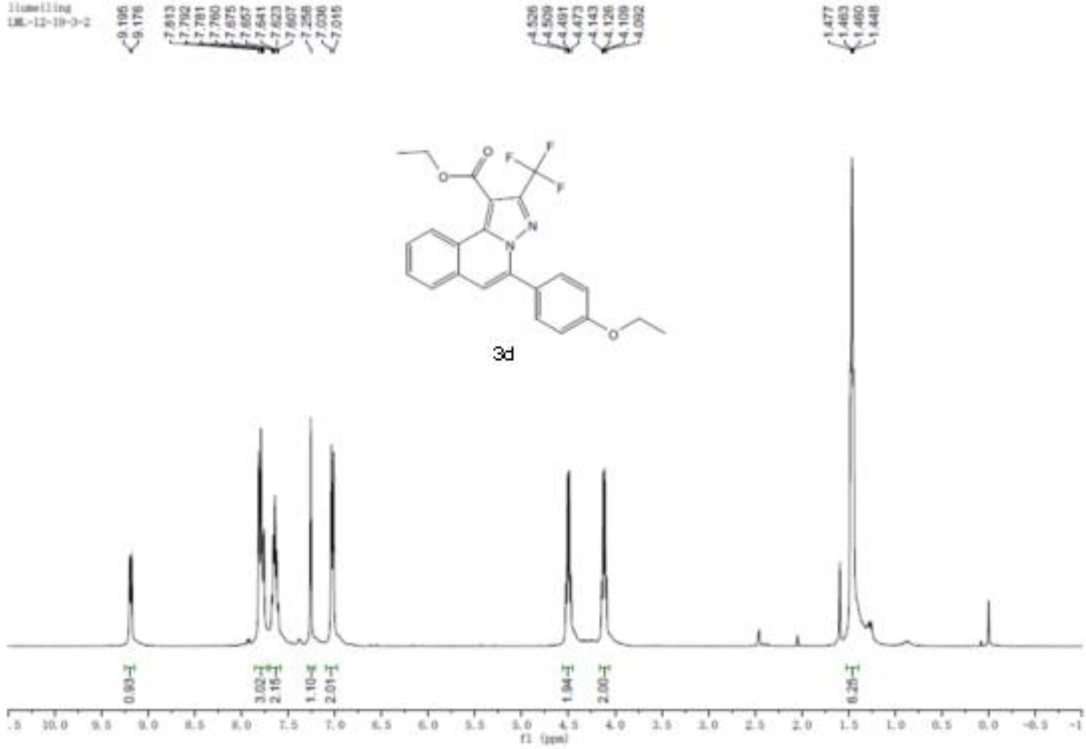

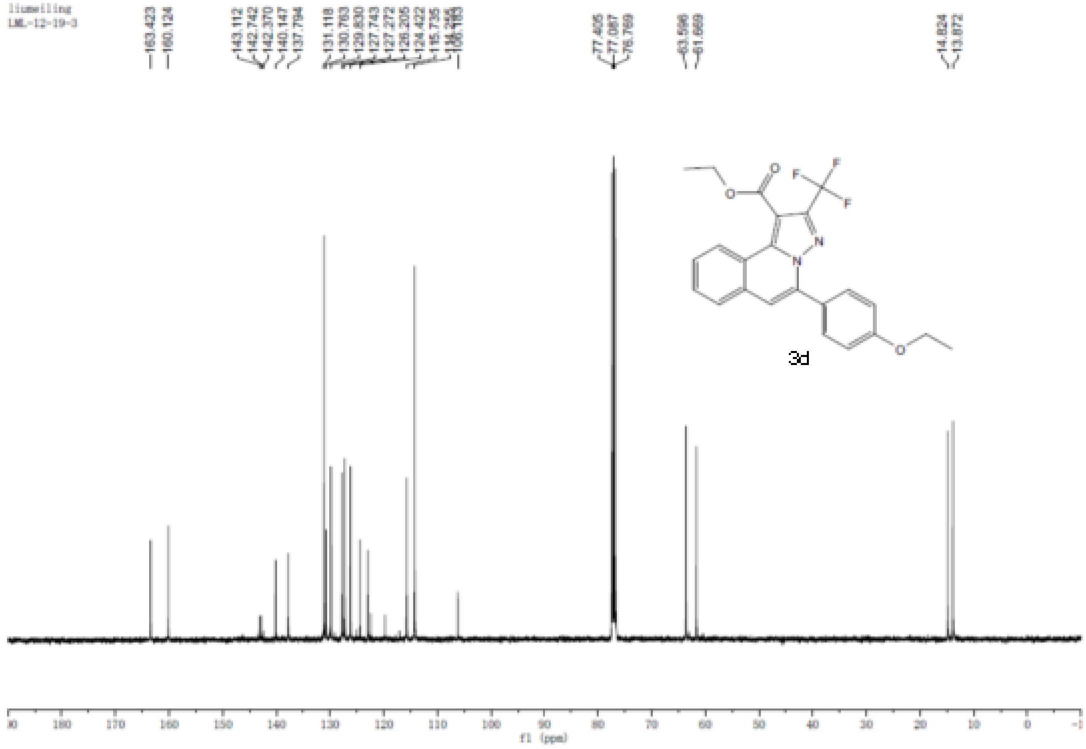

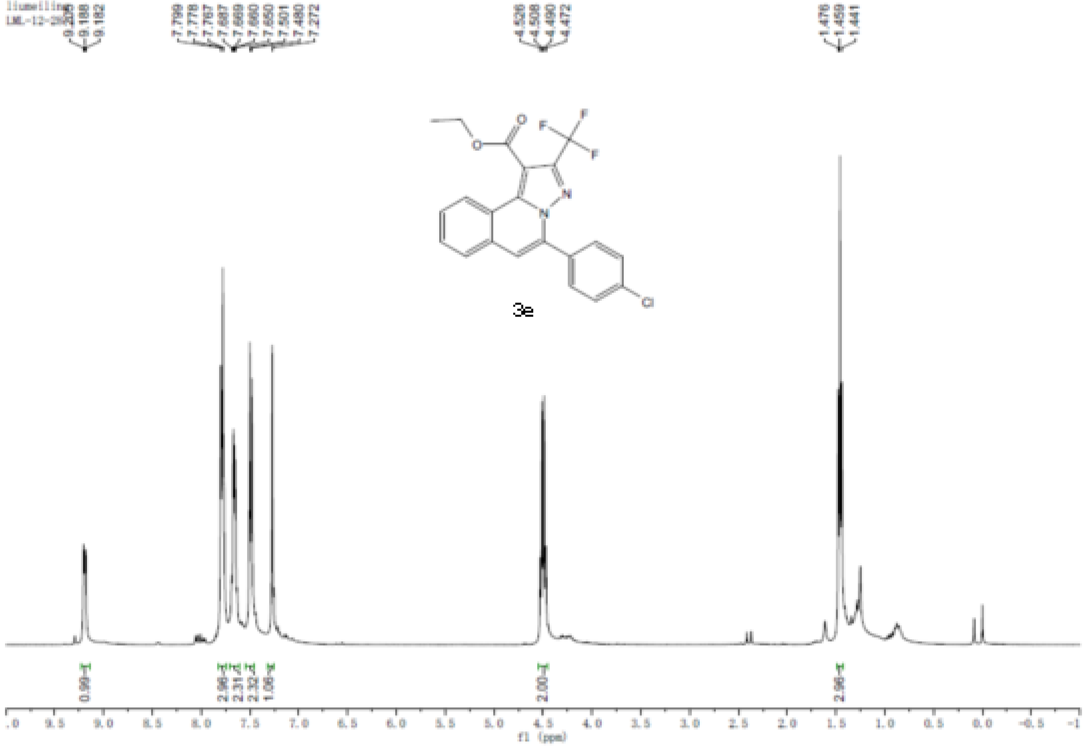

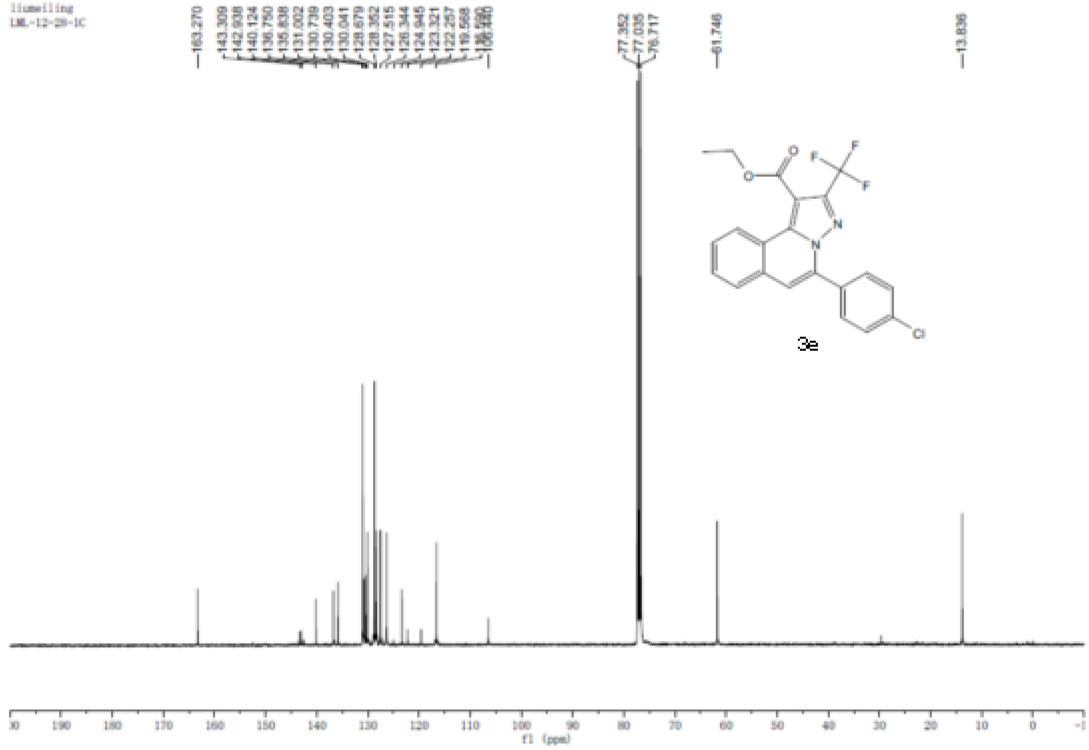

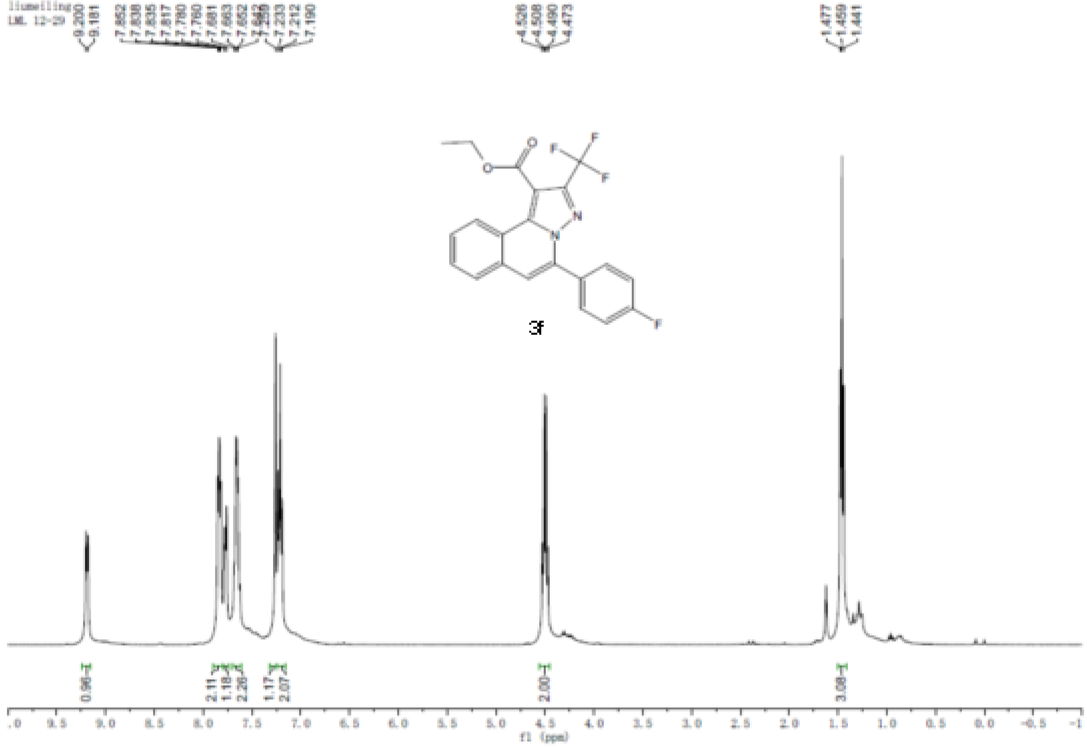
liumeiling
LML 12-29
9.200
9.181
7.852
7.838
7.835
7.817
7.780
7.760
7.681
7.663
7.652
7.233
7.212
7.190
4.526
4.508
4.490
4.473
1.477
1.459
1.441
3f
0.96
2.11
1.18
2.26
1.17
2.07
2.00
3.08
f1 (ppm)

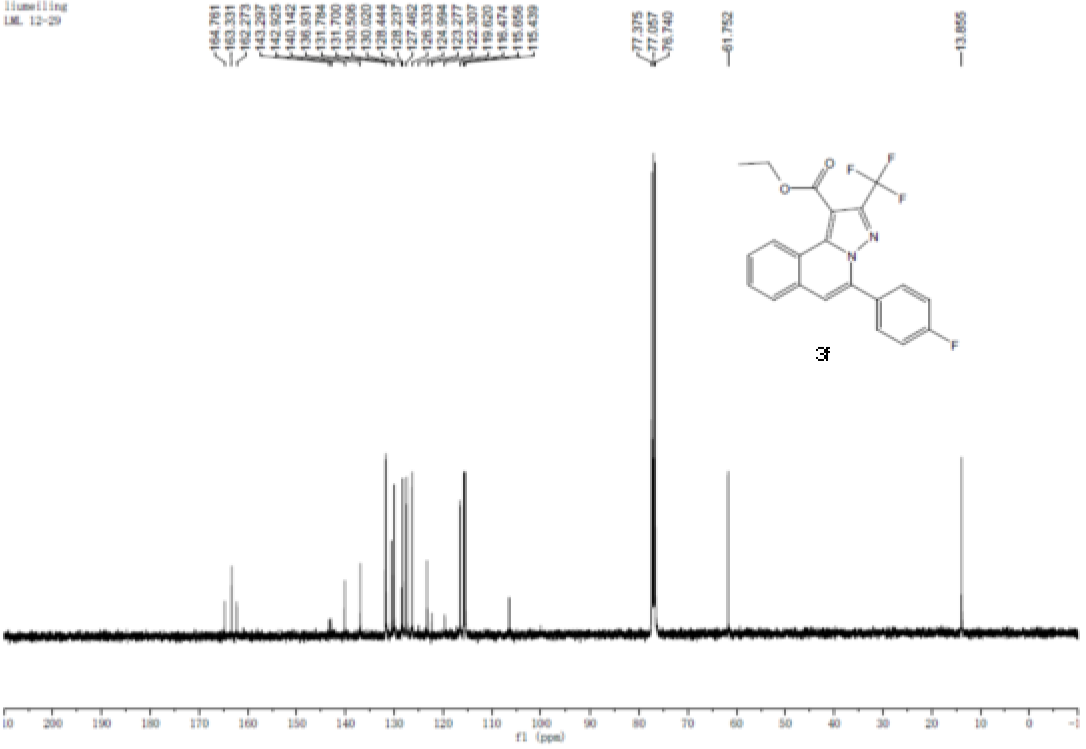
liumeiling
LML 12-29
164.761
163.331
162.273
143.297
142.925
140.142
136.931
131.784
131.700
130.506
130.020
128.444
128.237
127.462
126.333
124.994
123.277
122.307
119.620
116.474
115.656
115.439
77.375
77.057
76.740
61.752
13.855
3f
f1 (ppm)

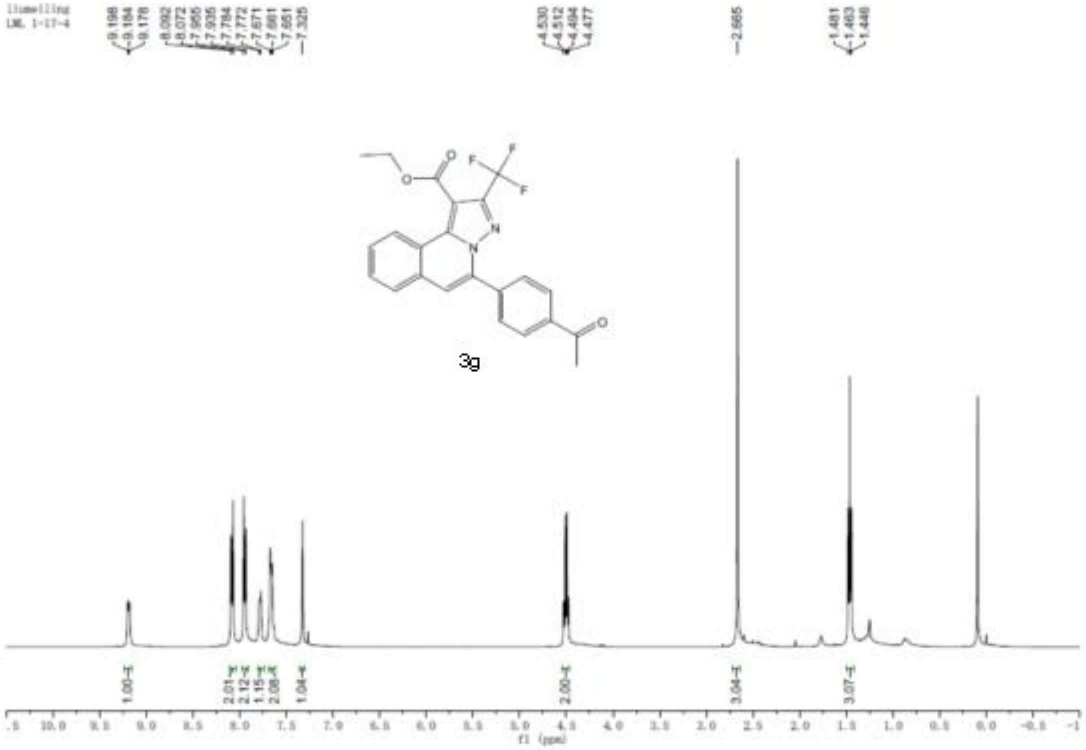

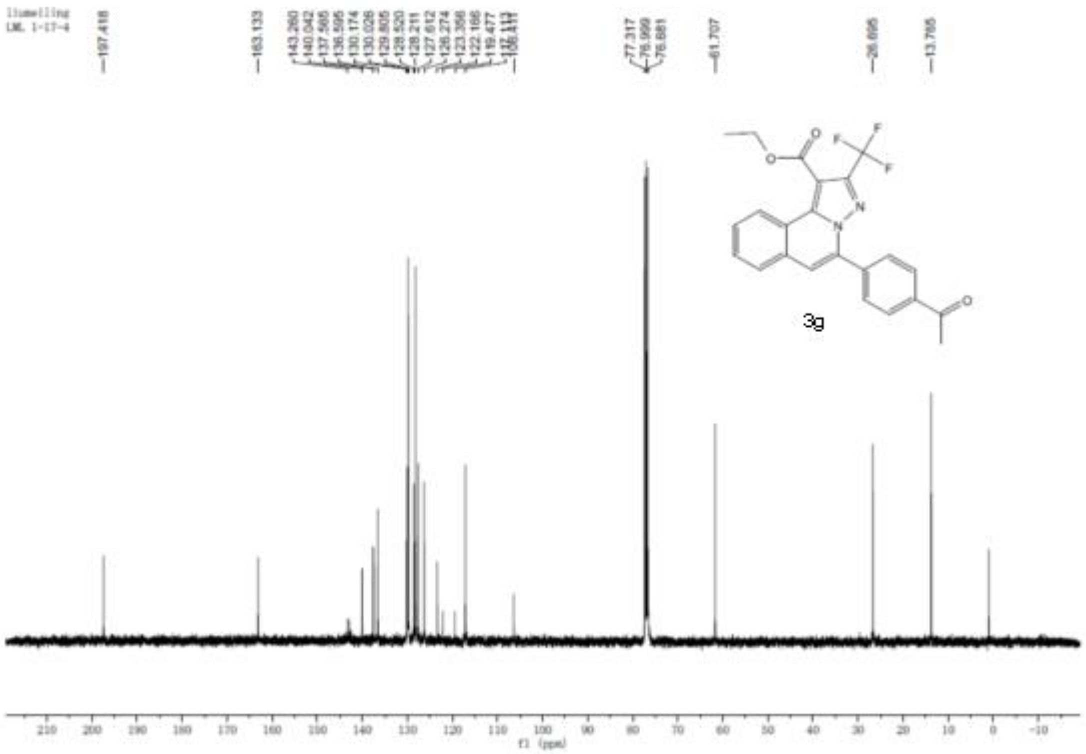

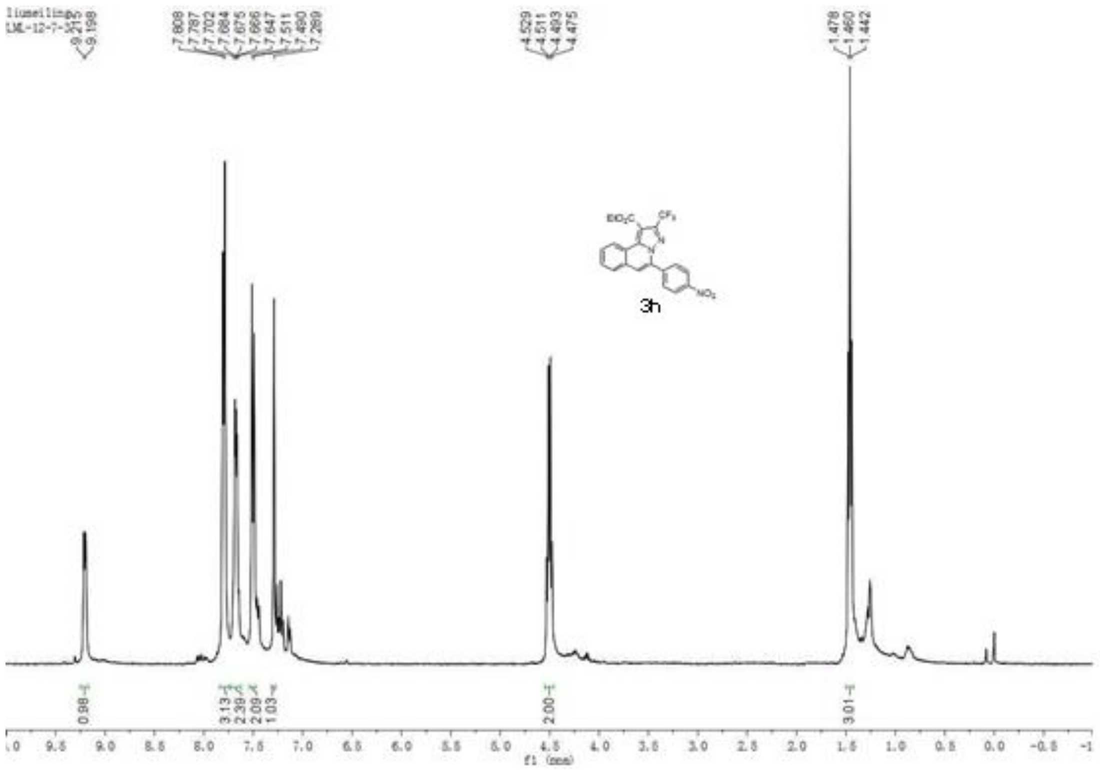
liumeiling
LML-12-7-3
EtO2C
CF3
NO2
3h
f1 (ppm)

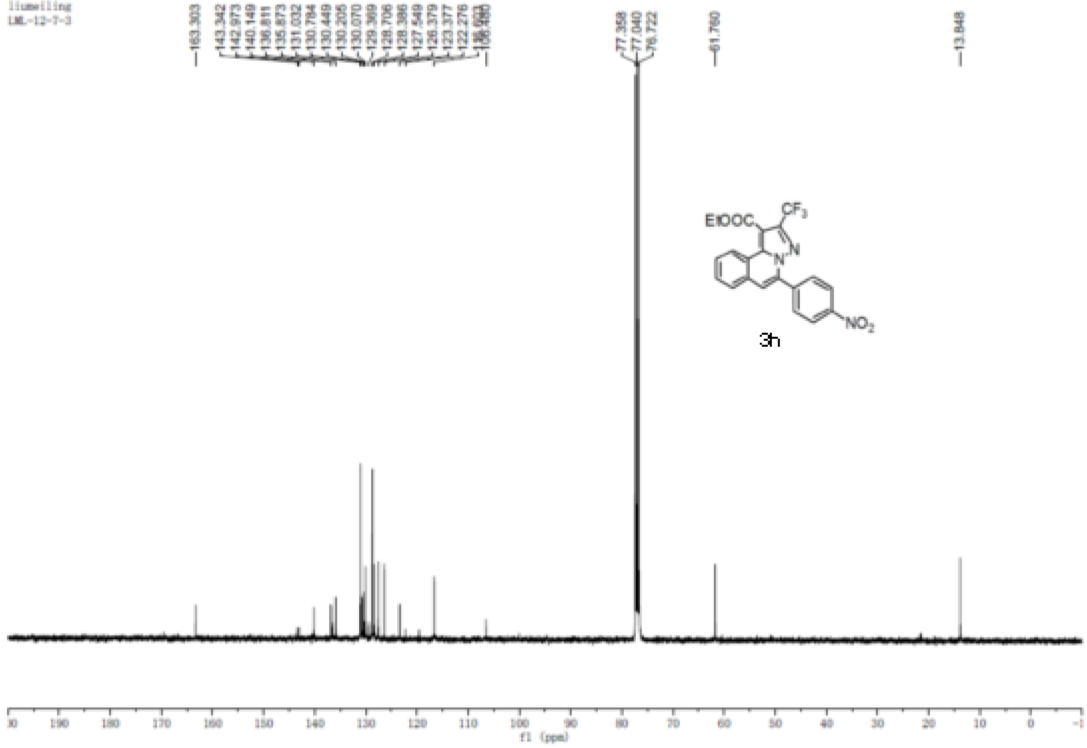
liumeiling
LML-12-7-3
EtOOC
CF3
N
N
NO2
3h
f1 (ppm)

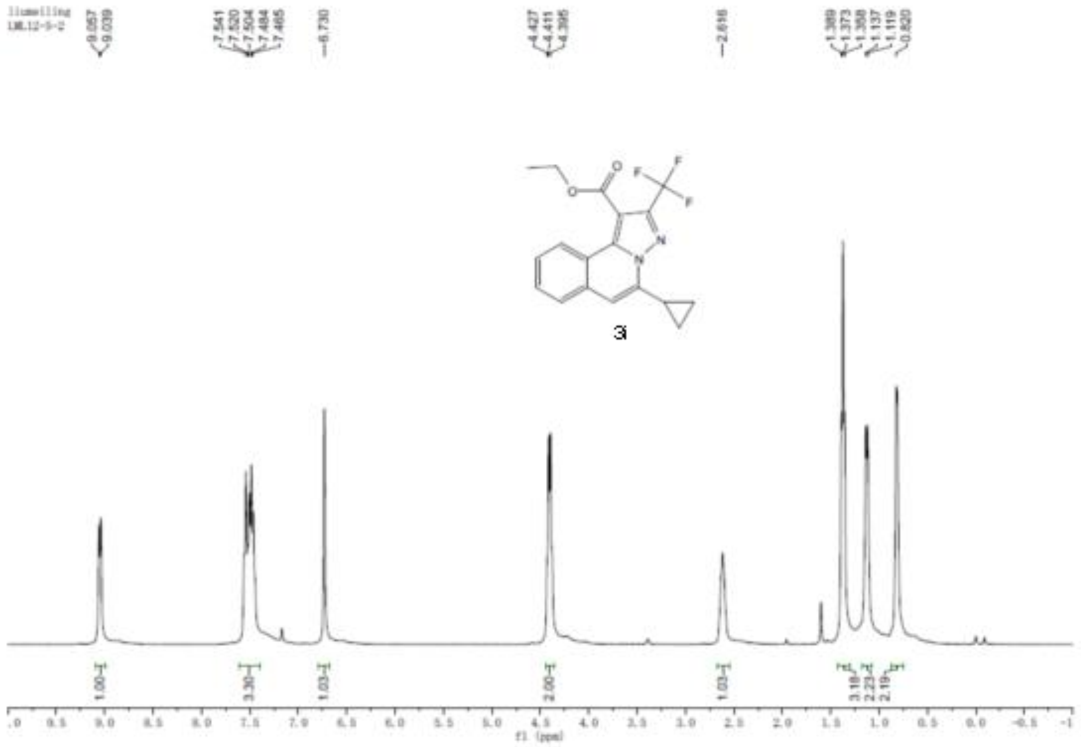
liumeiling
LML12-5-2
9.057
9.039
7.541
7.520
7.504
7.484
7.465
6.730
4.427
4.411
4.395
2.616
1.389
1.373
1.358
1.137
1.119
0.820
3i
1.00
3.30
1.03
2.00
1.03
3.18
2.23
2.19
f1 (ppm)

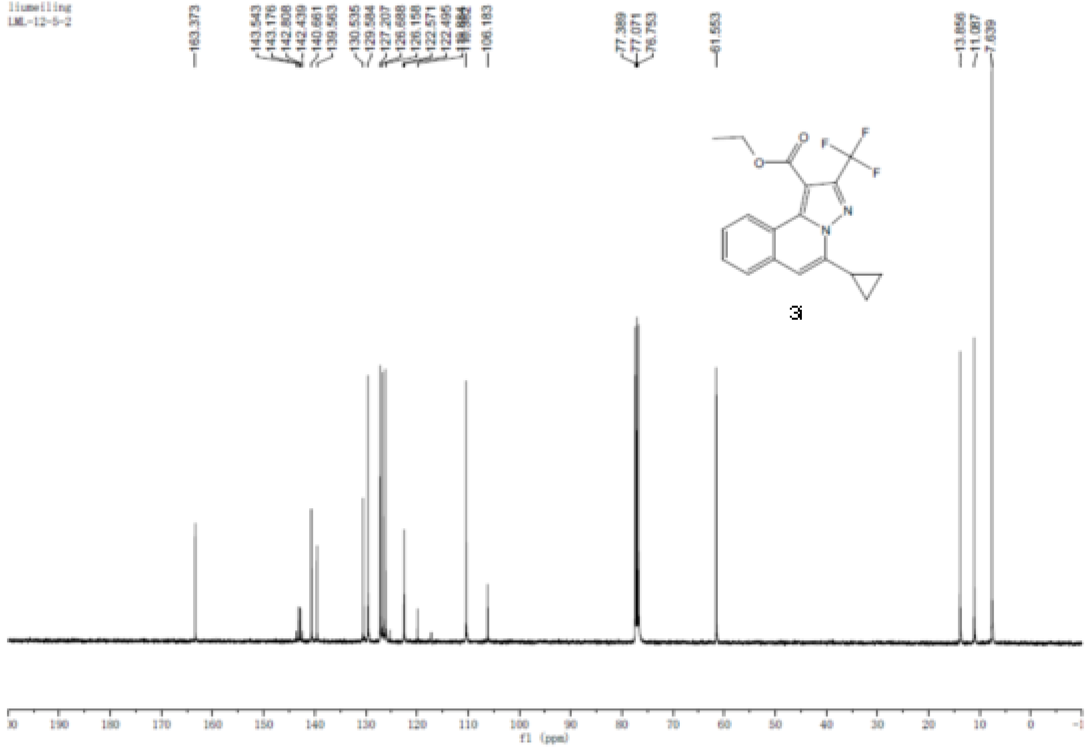
liumeiling
LML-12-5-2
163.373
143.543
143.176
142.808
142.439
140.661
139.563
130.535
129.584
127.207
126.688
126.158
122.571
122.495
106.183
77.389
77.071
76.753
61.553
13.856
11.087
7.639
3i
f1 (ppm)

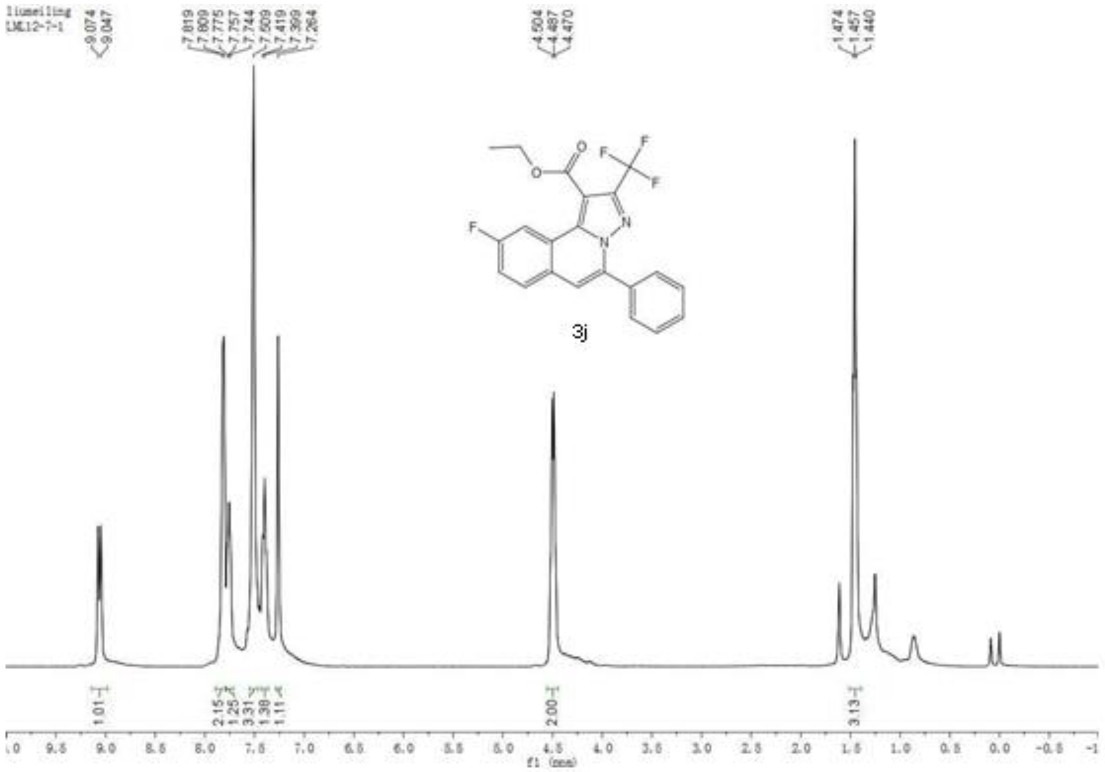

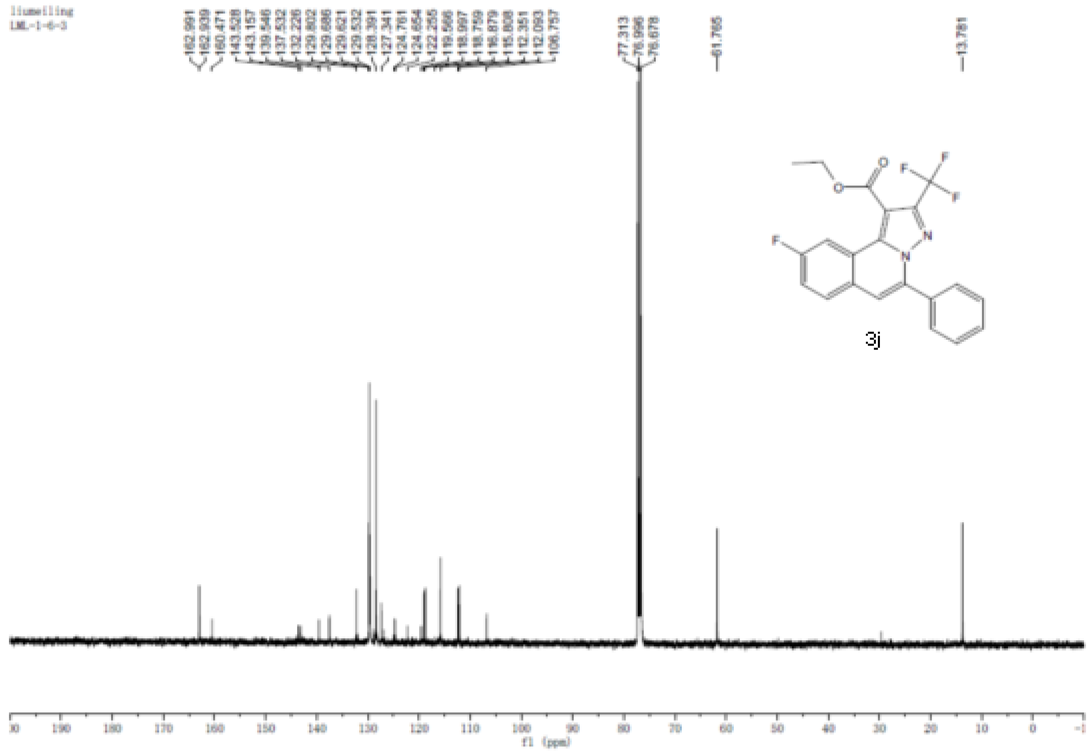

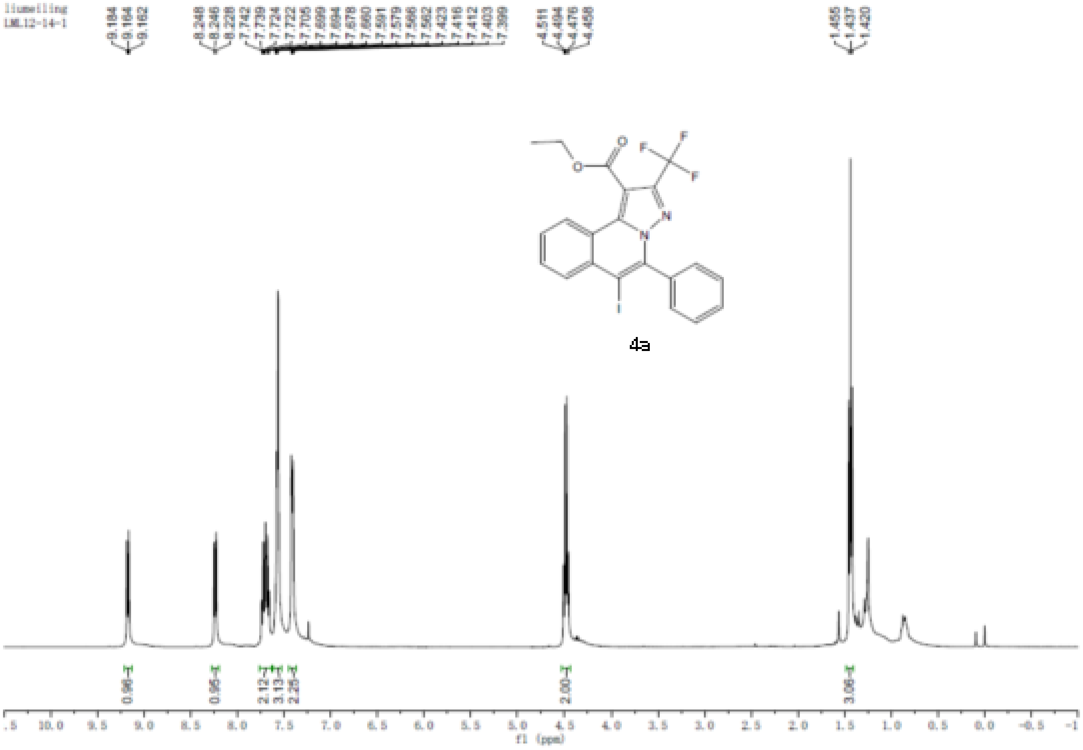

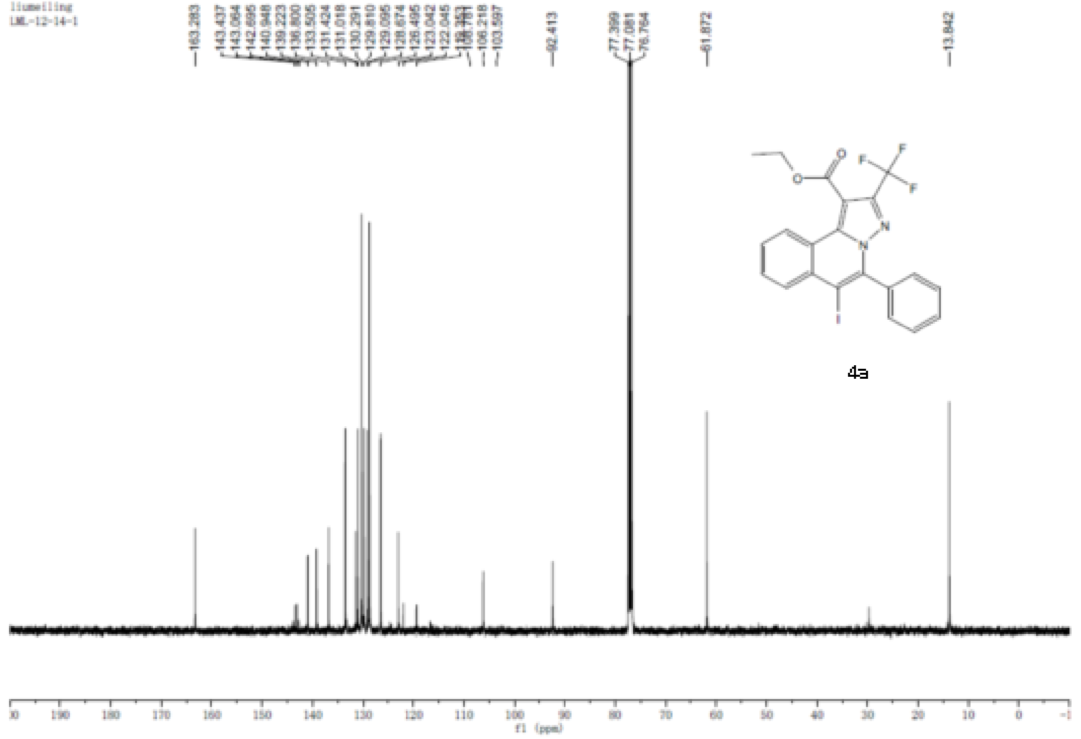

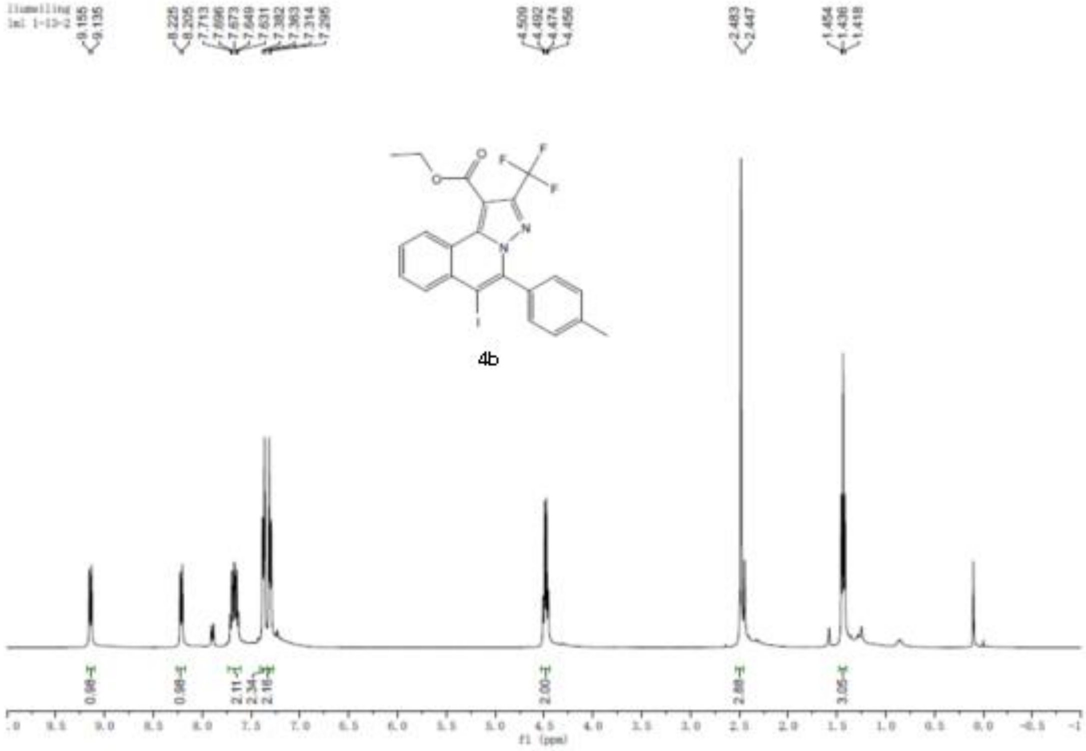

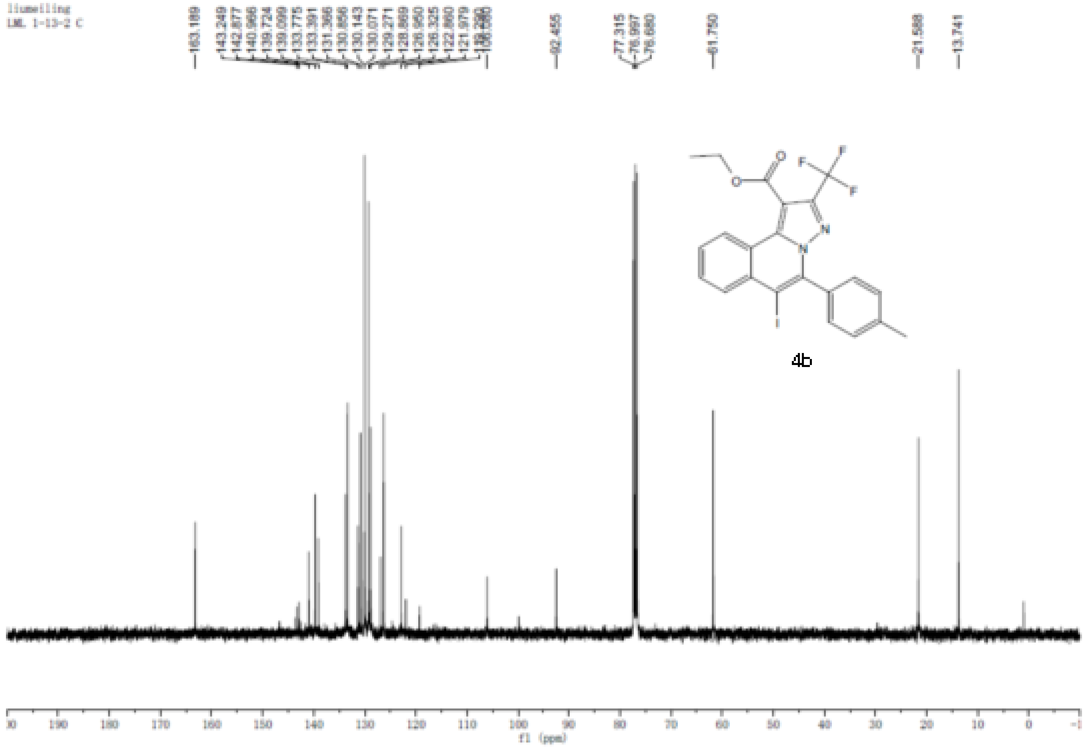

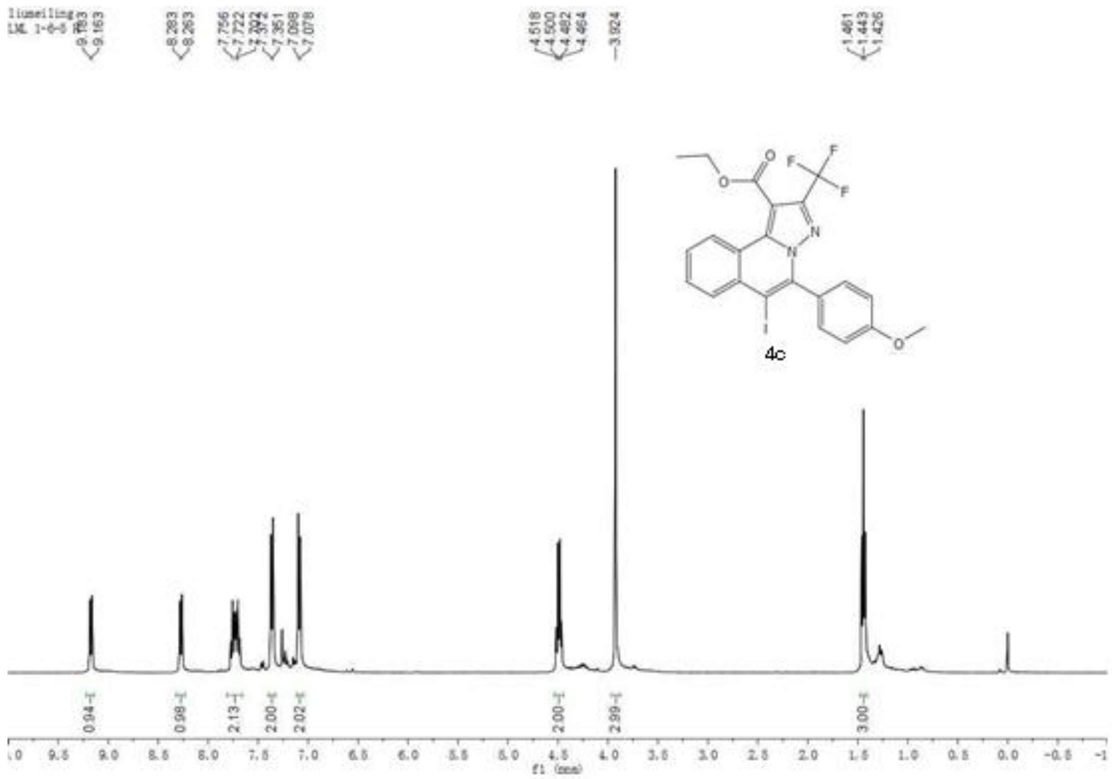

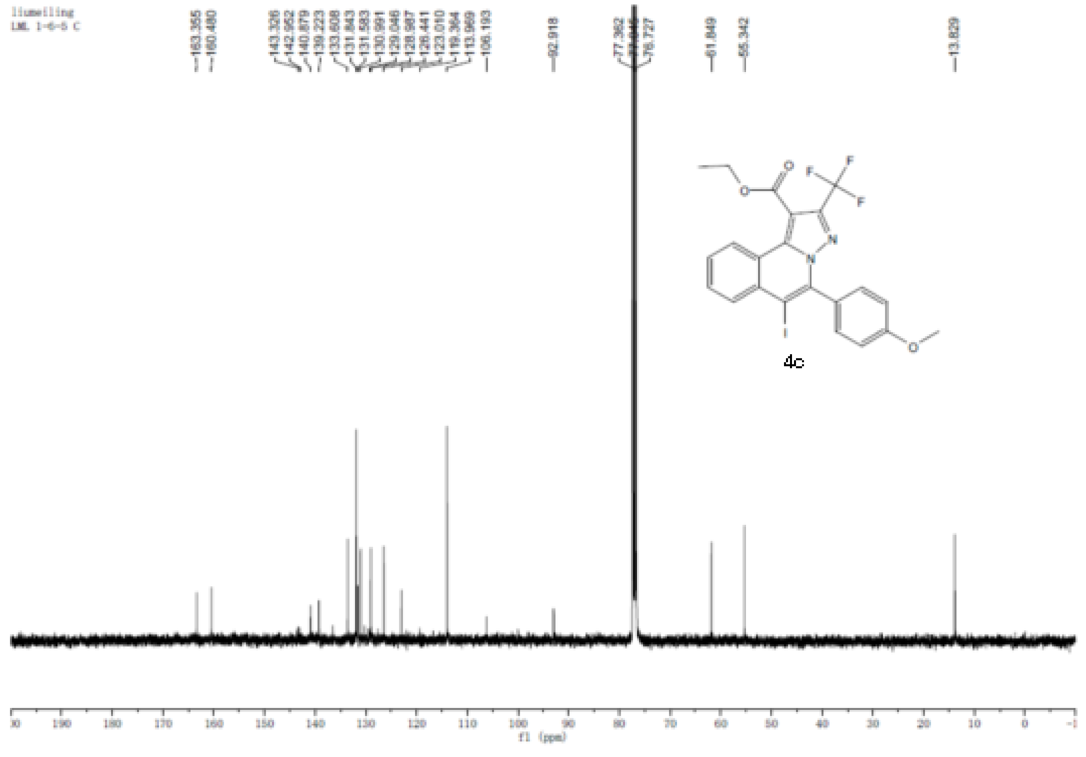

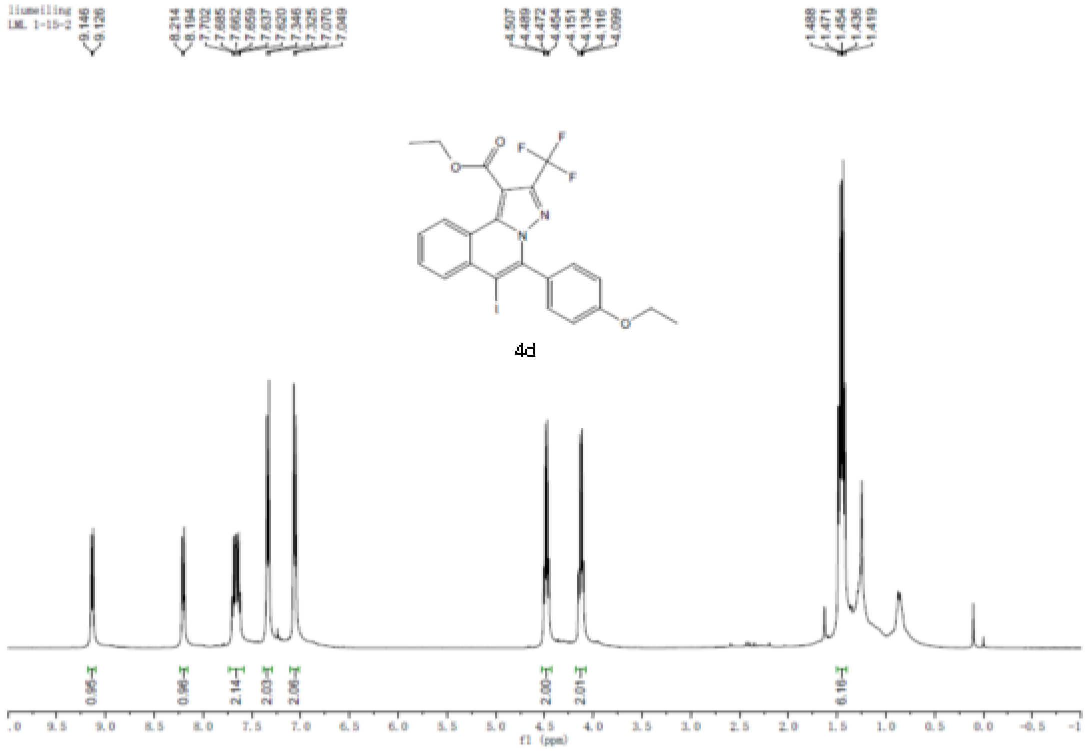
liumeiling
LML 1-15-2
9.146
9.126
8.214
8.194
7.702
7.685
7.662
7.659
7.637
7.620
7.346
7.325
7.070
7.049
4.507
4.489
4.472
4.454
4.151
4.134
4.116
4.099
1.488
1.471
1.454
1.436
1.419
4d
0.95
0.98
2.14
2.03
2.06
2.00
2.01
6.16
f1 (ppm)

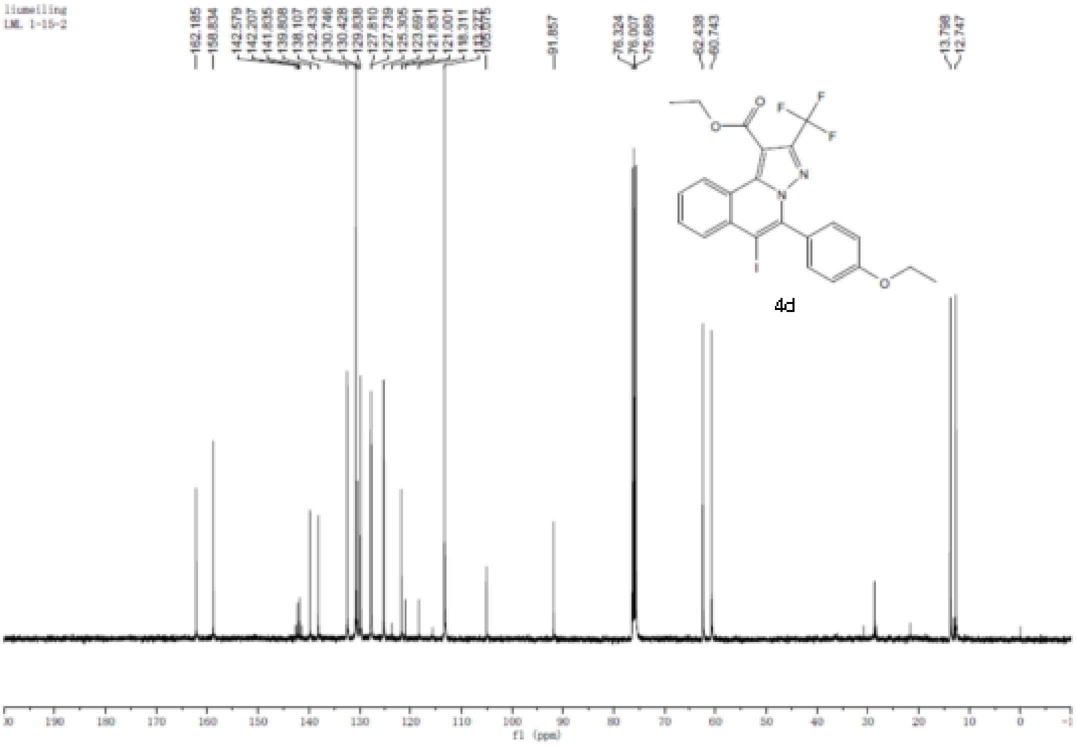
liumeiling
LML 1-15-2
162.185
158.834
142.579
142.207
141.835
139.808
138.107
132.433
130.746
130.428
129.838
127.810
127.739
125.305
123.691
121.831
121.001
118.311
76.324
76.007
75.689
62.438
60.743
91.857
13.798
12.747
4d
f1 (ppm)

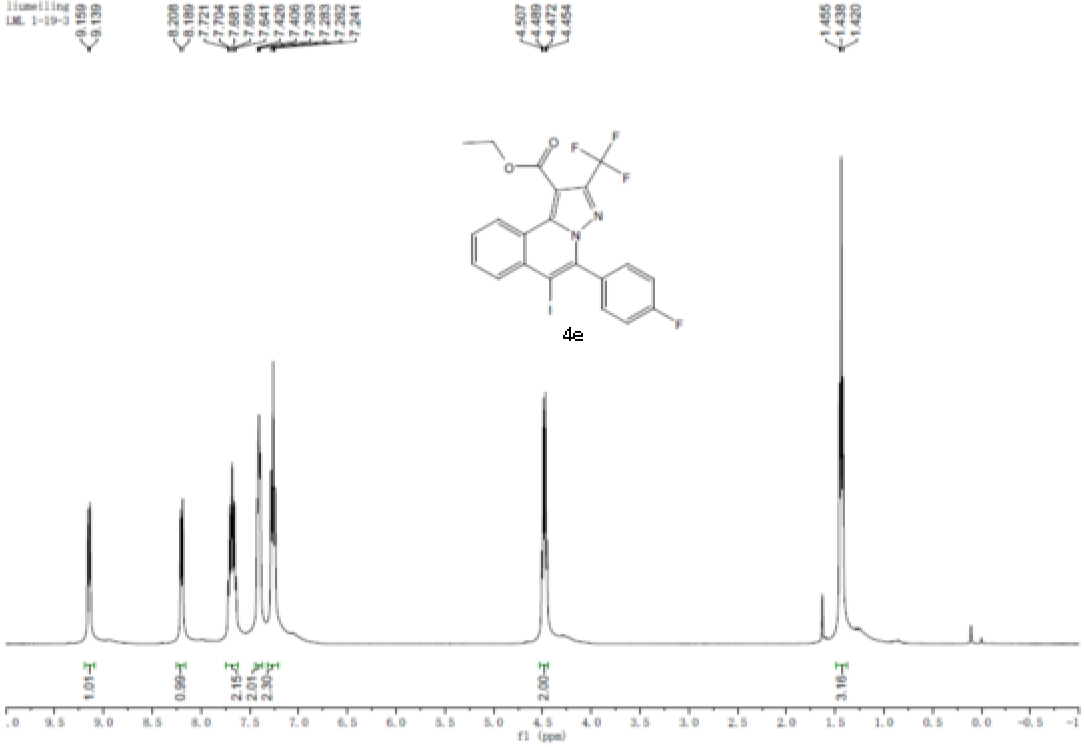
liumeiling
LML 1-19-3
9.159
9.139
8.208
8.189
7.721
7.704
7.681
7.659
7.641
7.426
7.406
7.393
7.283
7.262
7.241
4.507
4.489
4.472
4.454
1.455
1.438
1.420
4e
1.01
0.99
2.15
2.01
2.30
2.00
3.16
f1 (ppm)

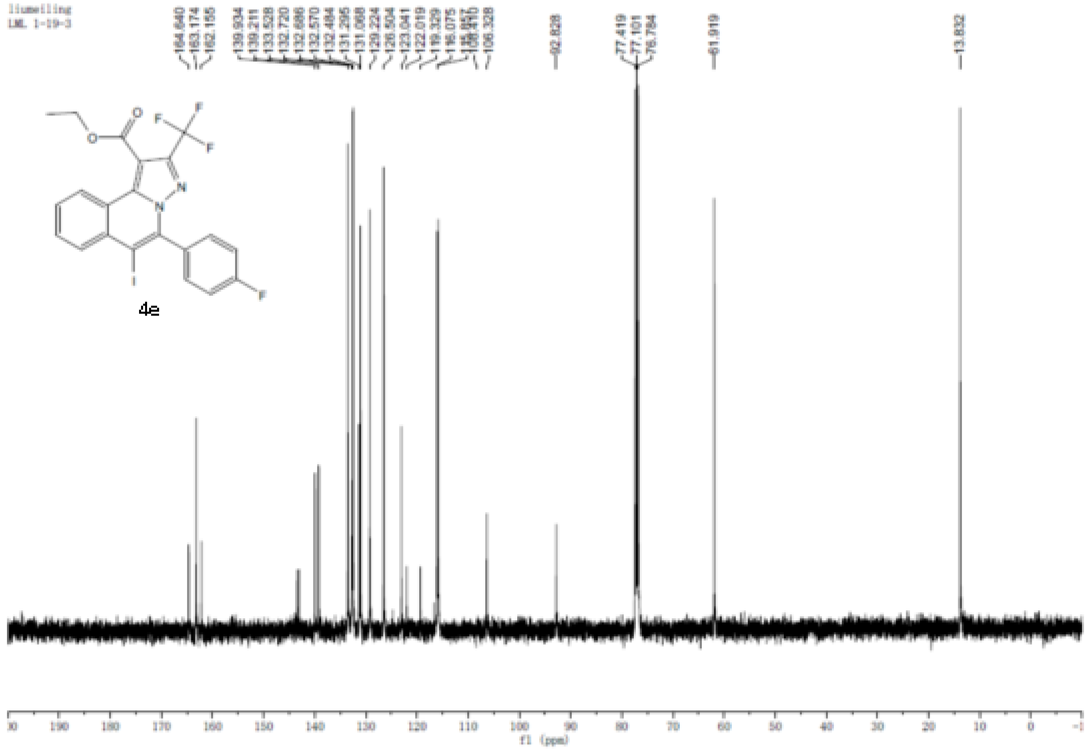
liumeiling
LML 1-19-3
164.640
163.174
162.155
139.934
139.211
133.528
132.720
132.686
132.570
132.484
131.295
131.068
129.224
126.504
123.041
122.019
119.329
116.075
106.328
92.828
77.419
77.101
76.784
61.919
13.832
4e
f1 (ppm)

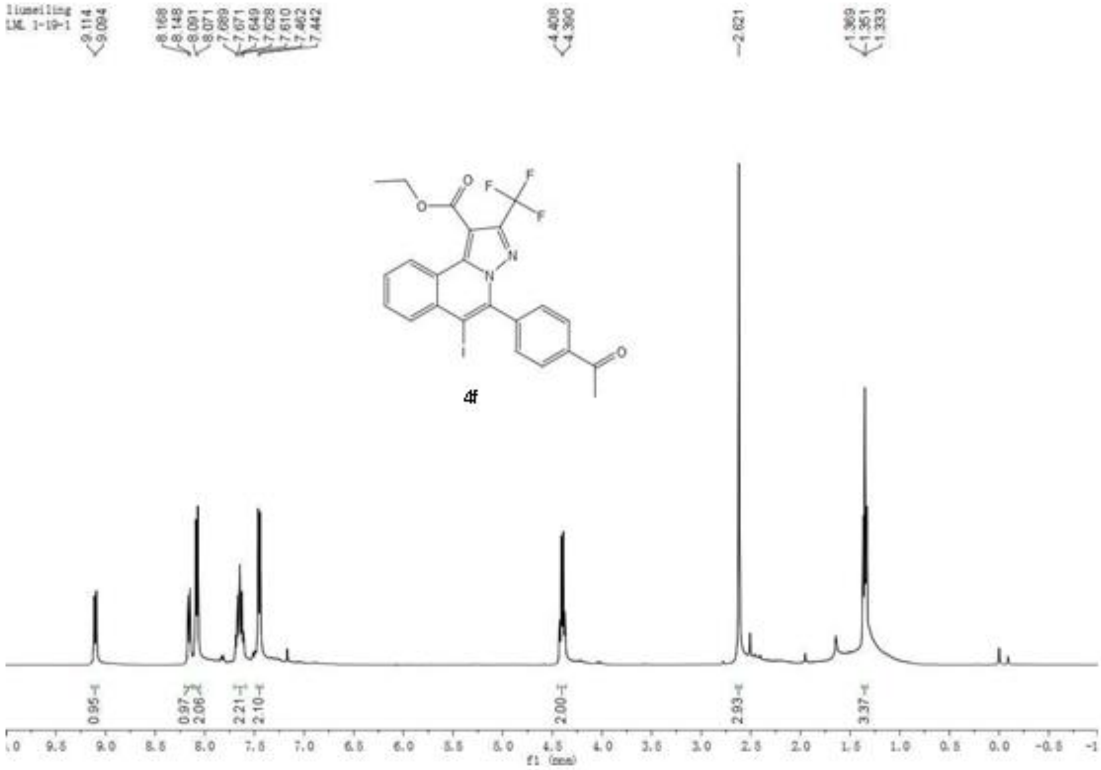
liumeiling
LML 1-19-1
4f
f1 (ppm)

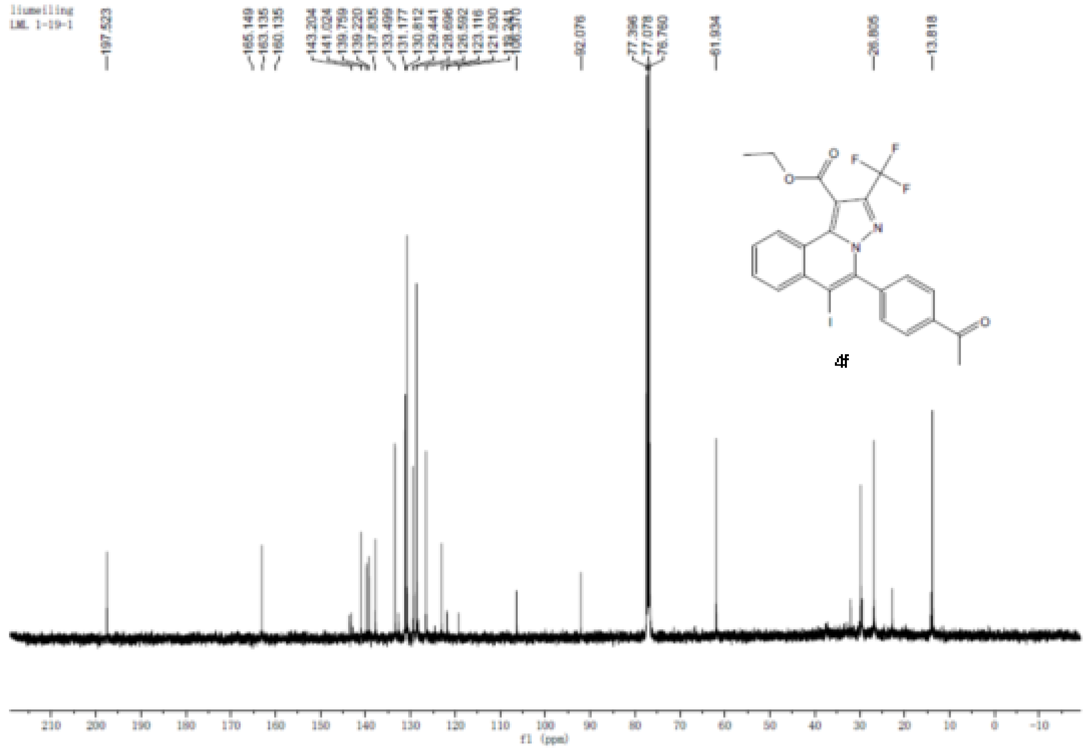
liumeiling
LML 1-19-1
4f
f1 (ppm)

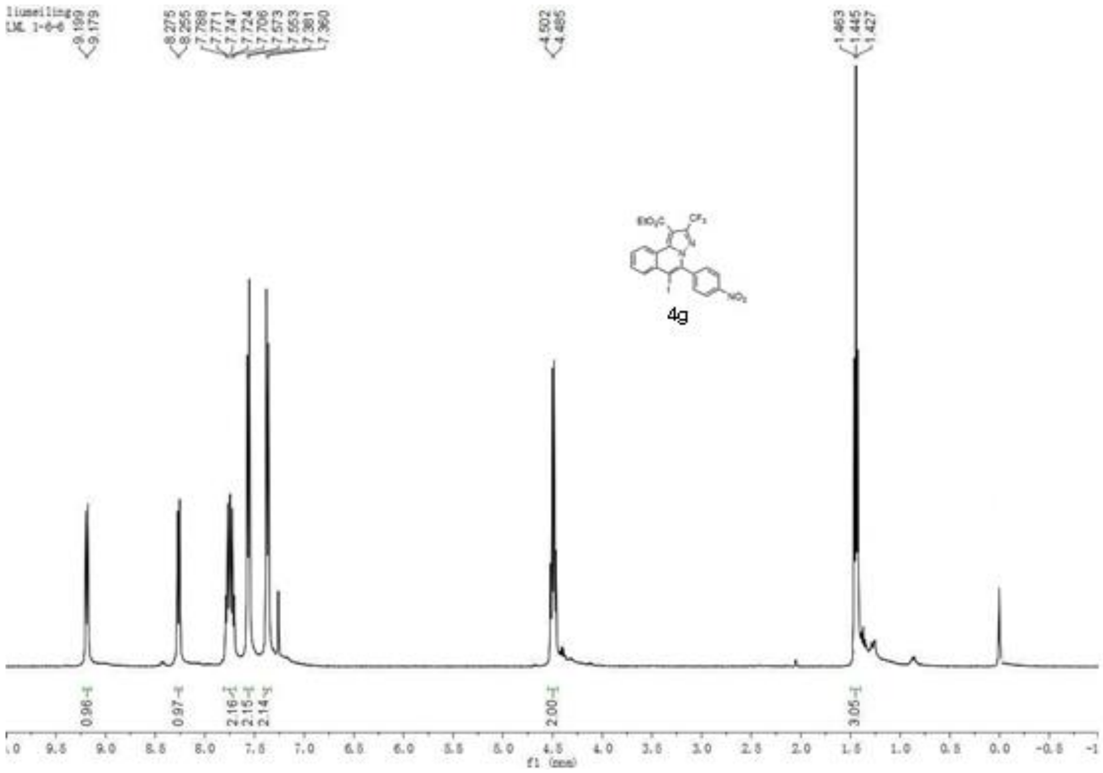
liumeiling
LML 1-6-6
9.199
9.179
8.275
8.255
7.788
7.771
7.747
7.724
7.706
7.573
7.553
7.381
7.360
4.502
4.485
1.463
1.445
1.427
4g
0.96
0.97
2.16
2.15
2.14
2.00
3.05
f1 (ppm)

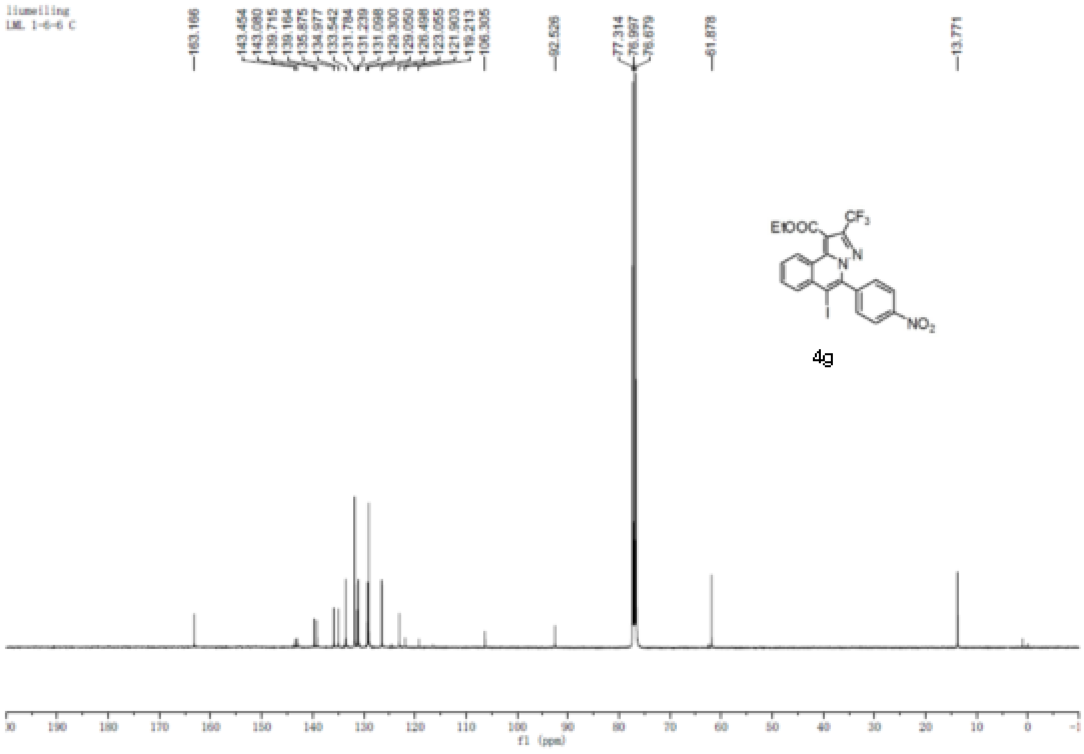
liumeiling
LML 1-6-6 C
163.166
143.454
143.080
139.715
139.164
135.875
134.977
133.542
131.784
131.239
131.098
129.300
129.050
126.498
123.055
121.903
119.213
106.305
92.526
77.314
76.997
76.679
61.878
13.771
EtOOC
CF3
N
NO2
4g
f1 (ppm)

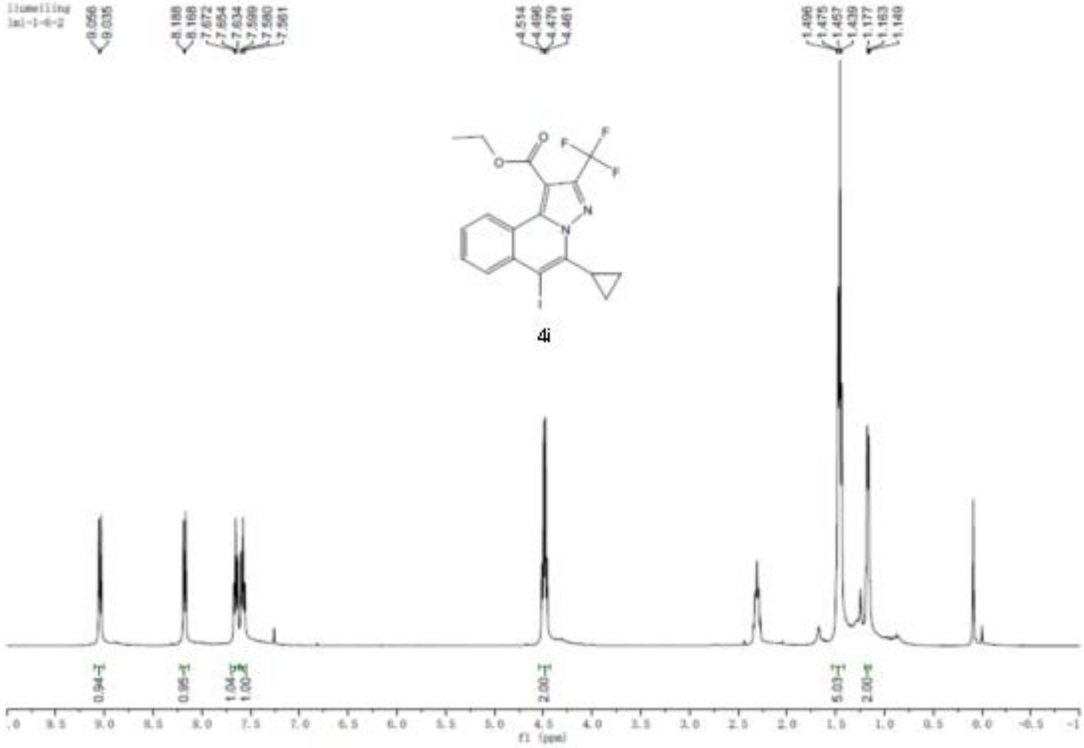

9.056
9.035
8.188
8.168
7.672
7.654
7.634
7.599
7.580
7.561
4.514
4.496
4.479
4.461
1.496
1.475
1.457
1.439
1.177
1.163
1.149
4i
0.94
0.95
1.04
1.00
2.00
5.03
2.00
f1 (ppm)

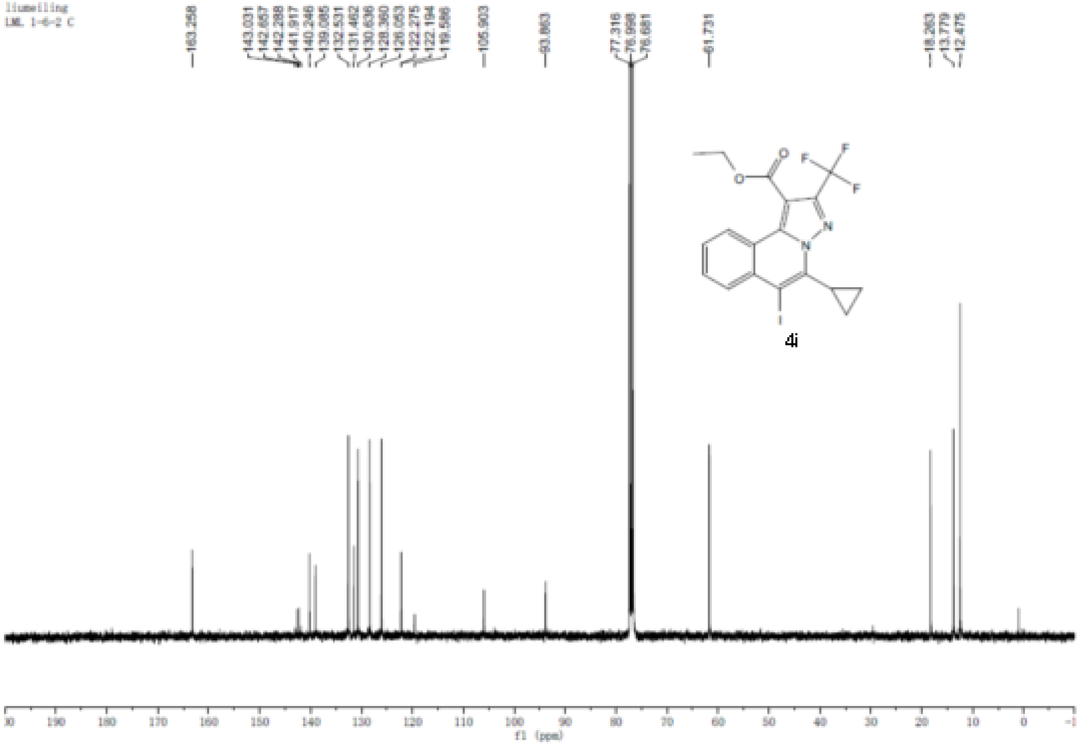

163.258
143.031
142.657
142.288
141.917
140.246
139.085
132.531
131.462
130.636
128.360
126.053
122.275
122.194
119.586
105.903
93.863
77.316
76.998
76.681
61.731
18.263
13.779
12.475
4i
f1 (ppm)

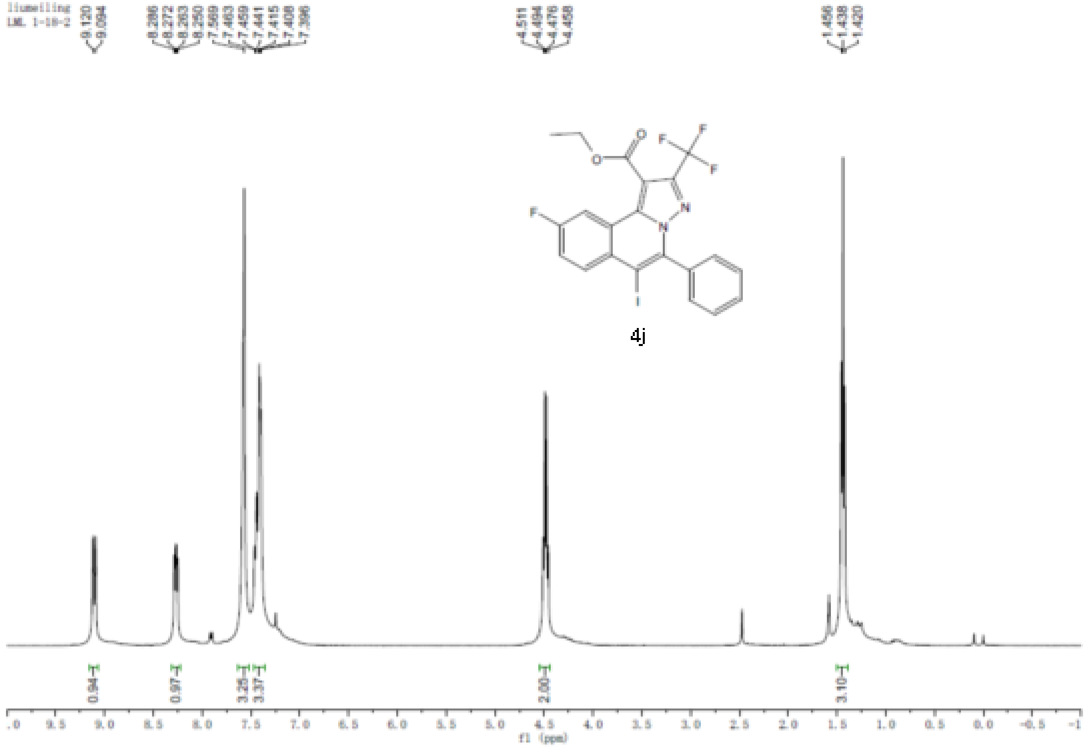

liumeiling
LML 1-18-2
9.120
9.094
8.286
8.272
8.263
8.250
7.569
7.463
7.459
7.441
7.415
7.408
7.396
4.511
4.494
4.476
4.458
1.456
1.438
1.420
4j
0.94
0.97
3.25
3.37
2.00
3.10
f1 (ppm)

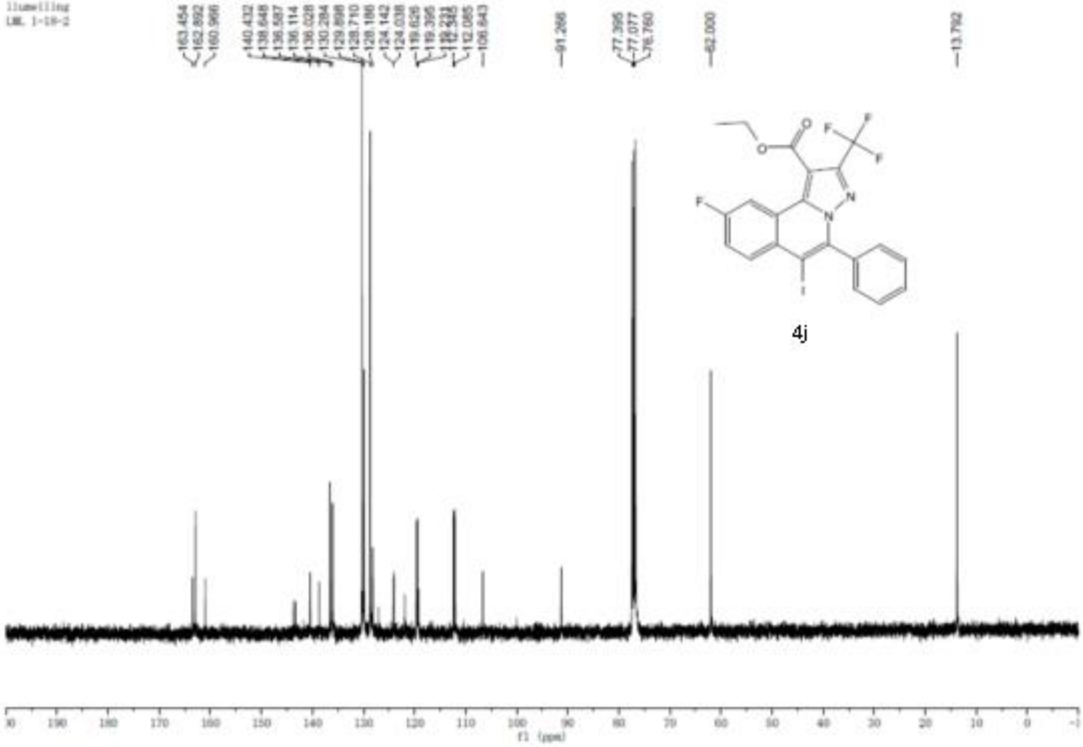

liumeiling
LML 1-18-2
163.454
162.892
160.966
140.432
138.648
136.587
136.114
136.028
130.284
129.898
128.710
128.186
124.142
124.038
119.626
119.395
112.085
106.643
91.266
77.395
77.077
76.760
62.000
13.792
4j
f1 (ppm)
